# A novel method to identify cooperative functional modules: study of module coordination in the *Saccharomyces cerevisiae *cell cycle

**DOI:** 10.1186/1471-2105-12-281

**Published:** 2011-07-12

**Authors:** Jeh-Ting Hsu, Chien-Hua Peng, Wen-Ping Hsieh, Chung-Yu Lan, Chuan Yi Tang

**Affiliations:** 1 Department of Computer Science, National Tsing Hua University, Hsinchu, 30013, Taiwan; 2 Departments of Resource Center for Clinical Research, Chang Gung Memorial Hospital, Taoyuan, 333, Taiwan; 3 Institute of Statistics, National Tsing Hua University, Hsinchu, 30013, Taiwan; 4 Department of Life Science, National Tsing Hua University, Hsinchu, 30013, Taiwan; 5 Institute of Molecular and Cellular Biology, National Tsing Hua University, Hsinchu, 30013, Taiwan; 6 Department of Computer Science and Information Engineering, Providence University, Taichung, 43301, Taiwan

## Abstract

**Background:**

Identifying key components in biological processes and their associations is critical for deciphering cellular functions. Recently, numerous gene expression and molecular interaction experiments have been reported in *Saccharomyces cerevisiae*, and these have enabled systematic studies. Although a number of approaches have been used to predict gene functions and interactions, tools that analyze the essential coordination of functional components in cellular processes still need to be developed.

**Results:**

In this work, we present a new approach to study the cooperation of functional modules (sets of functionally related genes) in a specific cellular process. A cooperative module pair is defined as two modules that significantly cooperate with certain functional genes in a cellular process. This method identifies cooperative module pairs that significantly influence a cellular process and the correlated genes and interactions that are essential to that process. Using the yeast cell cycle as an example, we identified 101 cooperative module associations among 82 modules, and importantly, we established a cell cycle-specific cooperative module network. Most of the identified module pairs cover cooperative pathways and components essential to the cell cycle. We found that 14, 36, 18, 15, and 20 cooperative module pairs significantly cooperate with genes regulated in early G1, late G1, S, G2, and M phase, respectively. Fifty-nine module pairs that correlate with Cdc28 and other essential regulators were also identified. These results are consistent with previous studies and demonstrate that our methodology is effective for studying cooperative mechanisms in the cell cycle.

**Conclusions:**

In this work, we propose a new approach to identifying condition-related cooperative interactions, and importantly, we establish a cell cycle-specific cooperation module network. These results provide a global view of the cell cycle and the method can be used to discover the dynamic coordination properties of functional components in other cellular processes.

## Background

Identifying the essential components in a specific biological process and detecting the associations among these components in response to various conditions are important for understanding cellular functions. Such components consist of interacting proteins, DNA, and other molecules such as complexes, pathways, and regulatory programs [1-4]. Therefore, a set of genes encoding proteins that are associated by functional related interactions, such as direct physical interactions between members of a complex, cascading interactions of a pathway, or regulatory interactions between a factor and it's targets, form a functional module to facilitate a specific cellular function [2-4]. To conduct a cellular process, module cooperation is necessary to properly facilitate signal transduction, regulation, and metabolism. This cooperation can be established by direct interactions among components (crosstalk) or through shared partners [5,6]. To adapt to changing environmental conditions, the formation of functional modules and interactions among these modules are likely to be dynamic and condition-specific. To sustain cellular activities upon changes in the extra- or intracellular environment, specific functional modules and interactions among modules are induced by a series of signaling and regulatory cascades [3,4,6-8]. For example, under low-nitrogen conditions, crosstalk is observed between two signaling pathways in *Saccharomyces cerevisiae*, the cAMP and MAPK pathways, which are both downstream of the small GTPase Ras. These pathways in turn control the cell surface glycoprotein Flo11 and are involved in invasive and filamentous growth [9,10]. Therefore, discovering dynamically assembling modules, associations among these modules, and their condition-specific functions are critical for understanding the mechanisms of a biological process.

Large amounts of yeast two-hybrid, DNA microarray, and other high-throughput data are now publicly available [11-14]. These datasets not only provide information related to gene function and direct interactions among genes, but they also enable the use of clustering-based methods to discover functional modules [3,15-20]. By applying clustering algorithms to different datasets, various types of functional modules, including protein complexes, co-regulated modules, and signaling and metabolic pathways, can be extracted. In addition, with datasets derived from specific experimental conditions, functional modules with special properties, such as evolutionarily conserved complexes and condition-related functional components, can also be found [3,17,19,21]. Based on the identified modules, researchers can use network measurement approaches to further analyze the properties of a module or to compare modules from different datasets to elucidate various biological characteristics [16,19]. Clustering-based approaches, however, only focus on module identification and do not consider the connectivity between modules. Therefore, these approaches do not readily provide information about associations between modules such as module cooperation.

Recently, several groups have developed approaches to discover coordinated relationships between pairs of modules and to establish more complete frameworks for various cellular processes [5,22,23]. One type of approach searches for crosstalk pathways that significantly interact. By measuring the number of protein-protein interactions among all possible pathway pairs from a database, such as BioCarta, the pathway pairs with a statistically significant number of protein interactions can be identified [23]. Another type of approach aims to select module pairs that are coordinated in their gene expression levels by using data from Gene Ontology (GO) and DNA microarrays [5,22]. Thus, these methods identify coordinated relationships that are co-regulated by common regulators or are co-expressed under specific conditions. Both types of approaches are suitable for characterizing the properties of module association.

Although the above-mentioned methods can be used to measure correlations between module pairs, they ignore interactions mediated by genes that associate with module pairs. These interactions are direct clues used to interpret the influence, function, and mechanisms of module cooperation and, importantly, to estimate the necessity of the cooperation between a module pair. Moreover, as the modules evaluated by these methods are previously-defined gene sets, it is difficult to identify dynamically assembled functional modules and correlations between modules in a specific condition. Therefore, tools still need to be developed to discover and study cooperating module pairs that function in important signal transduction, regulatory and metabolic reactions under specific conditions.

In this paper, we propose an approach to study module cooperation. We identified cooperating module pairs by searching for functional module pairs that significantly correlate with genes with important functions and genes that mediate communication between functional components of a process. To evaluate our approach, we also analyzed the functions, cooperating genes, and mechanisms of each identified module pair. Using the yeast cell cycle as an example, we identified cooperating module pairs and predicted the mediators and interactions that are important for module cooperation in each phase of the cell cycle. The yeast cell cycle is divided into four phases: G1, S (synthesis), G2, and M (mitosis). During this cycle, a cell duplicates and divides into two daughter cells through a series of regulatory events and checkpoint mechanisms. Cell cycle-specific components dynamically assemble and interact with specific factors to control progression through the cell cycle. For example, in G1 phase, the major regulator Cdc28 combines with G1 cyclins and associates with other G1-specific transcription factors, such as the SBF complex (Swi4/Swi6), to regulate G1/S-specific genes and prepare the cell for DNA replication [24,25]. In S phase, specific component coordination appears to promote DNA replication, bud emergence, SPB duplication, and SPB separation [26]. In G2 and M phases, Cdc28 and B-type cyclins form complexes that induce chromosome condensation, spindle elongation, and nuclear division [27]. In addition, to ensure that events of the cell cycle finish completely, checkpoint mechanisms coordinate multiple pathways to control progression through the cell cycle [28]. Due to its complex regulation and the dynamic interactions of its components, studying the cell cycle requires a systematic approach that analyzes cooperation among functional components.

Rather than considering only one type of data, our approach provides a platform that allows interaction and expression data to be integrated. The expression data provide information about dynamic correlations among genes in the yeast cell cycle, and the interaction data suggest possible interactions among genes. This information can be used to predict genes and interactions that may function in the yeast cell cycle. Advantages of combining heterogeneous data were demonstrated by the studies of functional association prediction. These approaches used a probabilistic model to combine expression correlations and physical interactions between genes measured from different experimental data sets [29-31]. The combined scores were used to establish a gene network to present the functional associations between genes and to predict gene function [29,31]. To identify functional modules and the cooperating pairs that directly interact with genes essential to the cell cycle, we used a different approach to combine information from protein-protein interactions, ChIP-chip data, and microarrays. We did not use combined association scores between genes to construct the gene network but instead used direct physical interactions to represent links among genes. However, information from expression correlation was used to measure the essentiality of genes to the cell cycle. Therefore, we can design an algorithm to search cooperating sub-networks (modules) based on the physical interaction network. In addition, we evaluated the importance of module cooperation and only reported module pairs that significantly influence the cell cycle process. To analyze the architecture and special properties of module cooperation in the cell cycle, the resulting module pairs were further used to construct a cooperative module network (CMN). This cooperative module network presents cell cycle-specific modules and cooperative associations between the modules.

To understand the functions and communication mechanisms of each cooperative association, we also predicted genes related to each cooperative association (correlated genes). Such genes could be regulators, signal communicators, regulated genes, or members of a protein complex. Based on interactions among these correlated genes and genes within the modules, we further inferred the functions and effects of the cooperative associations in the cell cycle. Thus, we used a gene set consisting of genes regulated in a specific phase of the cell cycle and regulators of each phase to verify and explore cooperating interactions of the identified module pairs functioning in specific signal transduction, regulation and other activities of the yeast cell cycle. Using this phase-regulated gene set, we predicted phase-related interactions and genes mediating cooperative associations in a specific phase and then discovered dynamic changes in these interactions during the cell cycle. Based on interactions of phase-specific regulators, we constructed relationship graphs for each phase of the cell cycle to identify possible crosstalk among modules through phase-specific regulators and to attempt to explain the roles of transcriptional regulators in controlling the cooperation of and connections between modules. These graphs present a dynamic view of the module interactions in the yeast cell cycle. By comparing graphs, we gained important insights into the changes in associations between the different functional modules.

## Results

**Figure 1:**
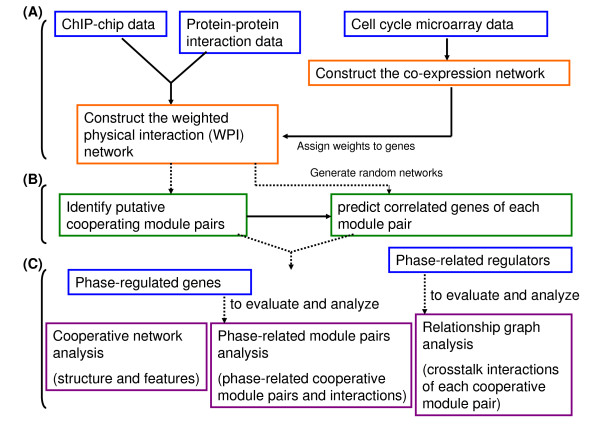
**Overview of the methodology developed in this study**. (A) Construction of the weighted physical interaction network (WPI network). The WPI network was established from protein-protein interaction (PPI) data, cell cycle expression data, and genome-wide location (ChIP-chip) data. Each node of the network indicates a gene, and each link indicates an interaction between two genes according to protein-protein interaction data and ChIP-chip data. The weight of each node was estimated by the correlations of gene pairs. We first established a co-expression network of nodes corresponding to genes and links corresponding to gene pairs with a Pearson correlation above 0.683 or below -0.683, and then we used the degree of each node in the network as its weight. (B) Identification of cooperative module pairs and correlated genes in each pair. Significantly cooperative module pairs were identified in the WPI network by a spanning algorithm. For each cooperative module pair, genes significantly correlated with both modules were also reported. (C) Cooperative module network and phase-related cooperation analyses. In this step, we evaluated our results and analyzed the structure and properties of the network generated by the cooperative module pairs, their interactions through correlated phase-regulated genes, and crosstalk mediated by phase-specific regulators. In addition, functionally cooperative module pairs and relationships in each phase of the cell cycle were inferred.

**Figure 2:**
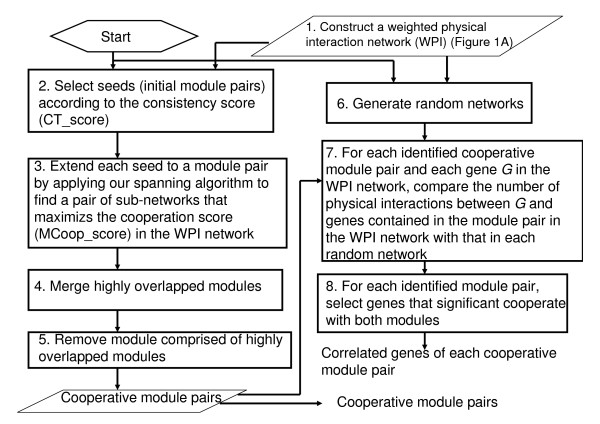
**The workflow of cooperative module identification and correlated genes identification**. The input of the process is the weighted physical interaction (WPI) constructed in the first step of our methodology (Figure 1A). Steps 1, 2, and 3 of the process are used to identify cooperative module pairs. In step 1, a WPI network is constructed. Nodes of the WPI network represent genes and links represent direct physical interactions between gene products. The weight of each node represents the degree of necessity of that gene in the cell cycle. In step 2, the consistency score (CT_score) of each gene pair is calculated. Gene pairs with CT_score above the 99^th ^percentile are selected as seeds. In step 3, each seed is extended to a module pair. The spanning algorithm is used to search a pair of sub-networks in the WPI network that contain the seed and maximize the module' cooperation score (MCoop_score). The step 6, 7, and 8 of the process are used to identify correlated genes of each cooperative module pair. In step 7, for each identified module pair and each gene *G *in the WPI network, the significance of the number of physical interactions between *G *and genes contained in the module pair in the WPI network is evaluated by random network (see Additional file 1 for more details). For each identified module pair, genes that are significant associated with both modules are selected in step 8.

To decipher how functional modules, such as protein complexes and signaling pathways, can cooperatively control the progress of the yeast cell cycle, we designed a method to study communication mechanisms among molecular components. The method developed in this work is outlined in Figure 1 and was divided into three steps: the first and second steps were designed to predict cooperative module pairs during cell cycle progression and to identify correlated genes that cooperate with each identified module pair (Figure 1A, B), and the final step was designed to evaluate our results and to analyze phase-related cooperative interactions among the identified module pairs (Figure 1C). To predict and analyze important cooperative associations among modules and genes, we combined a wide range of experimental data, including gene expression profiles, protein-protein interactions and ChIP-chip data. These data were used to construct a weighted physical interaction network (WPI network). Nodes of the WPI network represent genes and physical interactions between gene products are presented by links. The weight of each node represents the degree of necessity for a particular gene in the cell cycle. The flowchart of cooperative module identification and correlated genes identification is shown in Figure 2.

### Functional module

As mentioned, a module is defined as a set of genes whose products are connected by functionally related physical interactions that perform a specific cellular function.

### Cooperative module pair

We defined a cooperative module pair as two modules that significantly cooperate in a cellular process with each other and/or certain functional genes. Cooperation of a pair of modules can depend on direct crosstalk interactions or cooperative interactions through another gene product such as cofactors or common targets. Based on our method, a cooperative module pair can be identified by searching two distinct sub-networks with a significant number of cross-links or number of common interactions to genes essential in the cell cycle in the weighted physical interaction network.

### Correlated genes

**Figure 3:**
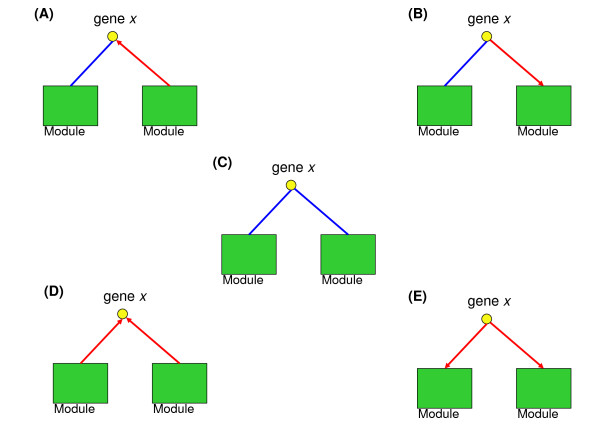
**Cooperation types of correlated genes**. Figure 3 shows the types of cooperative correlations between a module pair and its correlated genes predicted by our method. We predicted genes that mediate cooperative interactions between a pair of modules by evaluating the significance of the number of direct physical interactions between each module of a module pair and genes in the weighted interactions network (WPI network) with random networks (Figure 2). We used a yellow circle to indicate a gene and a green box to indicate a module. Consider a gene *x *in the WPI network and an identified module pair. If the number of protein-protein interactions (or regulatory interactions from ChIP-chip data) between *x *and a module is significant, the association was presented by a blue undirected line (or a red directed line) between the circle and a box. We identified five types of correlative associations: (A) A significant number of undirected links between *x *and genes in one of the modules and a significant number of directed links from genes in the other module to *x *in the WPI network. (B) A significant number of undirected links between *x *and genes in one of the modules and a significant number of directed links from *x *to genes in the other modules in the WPI network. (C) A significant number of undirected links between *x *and genes in each of the two modules in the WPI network. (D) A significant number of directed links from genes in each of the two modules to *x *in the WPI network. (E) A significant number of directed links from *x *to genes in each of the two modules in the WPI network.

We defined the correlated genes of a cooperative module pair as genes which have a significant amount of direct physical interactions to both modules (i.e. genes that significantly cooperate with both modules of a module pair). Cooperative interactions of module pairs can potentially be mediated by these correlated genes. Correlated genes were identified for each cooperative module pair (see Figure 2 and Additional file 1 for details). We predicted five types of significantly correlated genes with regard to cooperative module pair modules *M1 *and *M2 *(Figure 3): (1) genes that significantly interact with genes in one of the modules via protein-protein interactions and are significantly regulated by genes in the other module; (2) genes that significantly interact with genes in one of the modules via protein-protein interactions and regulate a significant numbers of genes in the other module; (3) genes that significantly interact with genes in *M1 *and *M2 *via protein-protein interactions; (4) genes that are simultaneously regulated by transcription factors in *M1 *and *M2*; and (5) genes that regulate significant numbers of genes in *M1 *and *M2*.

#### Identification of cooperative functional module pairs in the cell cycle

Module cooperation can have significant effects on the progression of a process because cooperative interactions that are mediated by genes in two modules can be required to activate or regulate genes with essential functions in that process. These cooperative effects could be achieved by transcriptional regulation, protein-protein interactions, protein phosphorylation, formation of a protein complex, or a combination of regulation and activation by genes from each module. To identify cooperative module pairs that are essential to cell cycle progression, we evaluated the possibility that any two modules cooperatively relate to cell cycle-associated genes by investigating the interactions (protein-protein interactions and regulatory interactions) that bridge both modules via other intermediate genes within or outside the two modules in a WPI network.

### Weighted physical interaction network (WPI network)

The WPI network is shown as a weighted graph in which nodes represent genes and links represent protein-protein interactions or regulatory interactions between gene products. Links can be used to infer functional modules and cooperation among those modules. The weight of each node represents the degree of necessity of that gene and was derived from the degree of the node in the aforementioned co-expression network (see the Methods section for more details). As significantly co-expressed genes tend to be functionally related, the Pearson correlation is a good scoring function used to evaluate the intensity of the functional correlation of a given pair of genes under specific conditions [15,32-34]. Furthermore, an essential role for most hub genes in a co-expression network has been shown to be more prevalent than with other genes in previous studies [35-38]; therefore, we used the number of co-expressed partners to estimate the probability that a gene is cell cycle-related. Based on the WPI network, we identified genes and their interactions that are likely to be significant and involved in the cell cycle.

If the cooperation of two modules is important in the cell cycle, genes associated with both modules by a significant number of cooperating interactions are possibly cell cycle-related. Thus, we designed the consistency score (CT_score) to measure the difference between the weights of genes correlated with a pair of modules and the weights of genes that are related to only one of the modules in the WPI network (Equation 1 in the Methods section). Higher numbers of cooperating interactions among a module pair and essential genes (within or outside the two modules) in the cell cycle process increase the consistency score of the module pair. In addition, to avoid local maxima and to incorporate genes possibly playing essential roles in the module but rarely linking to genes outside the module, we designed the mediation score (CoopMed score; Equation 3 in the Methods section) to incorporate genes that mediate interactions among genes within the module but has a few links to genes outside the module.

Finally, we designed the modules' cooperation score (MCoop_score; Equation 4 in the Methods section) to measure both the consistency score (CT_score; Equation 1 in the Methods section) of a module pair and the mediation score (CoopMed; Equation 3 in the Methods section). The cooperation score was our scoring function to estimate the possibility and importance of the cooperation of a module pair. A method that was designed to identify cooperative module pairs essential for the yeast cell cycle was illustrated in Figure 1A, B and 2 (see the Method section for more details).

#### Structure and properties of the cooperative module network (CMN)

**Figure 4:**
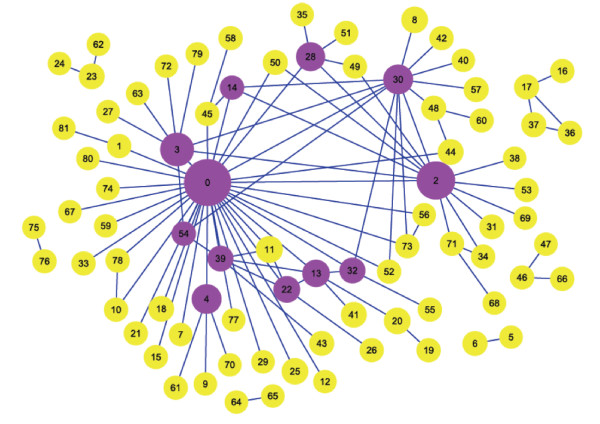
**The cooperative module network**. The cooperative module network (CMN) was constructed from the identified significantly cooperative module pairs. Modules are represented as circles and 101 identified associations between modules are represented as undirected links. The size of the circles is proportional to the number of genes within the modules. Purple circles indicate modules that appear in greater than or equal to four cooperative module pairs (Table 1). Genes contained in each module are listed in Additional file 2. Functions annotated from Gene Ontology (GO) are listed in Table 1 and Additional file 3. Most modules, especially modules presented as purple circles, were annotated as cell cycle related functions with significant *p*-values. Essential regulators that control cell cycle progression were also identified in functionally corresponding modules. For example, module 0 (response to DNA damage stimulus) contains *RAD53*; module 30 (cell morphogenesis) contains *CLN4*; module 3 (mitosis) contains *MAD1*; module 14 (regulation of transcription during G2/M-phase) contains *FKH1*; module 32 (regulation of cell division) contains *CDC28*.

After merging overlapped modules and the removal of module pairs comprised of highly overlapped modules, 101 cooperative module pairs and 82 functional modules containing three or more genes were identified (Figure 4; see Additional file 2 for results). In Figure 4, we generated a node to represent each of the 82 modules and 101 undirected links to indicate the identified cooperative relationships between modules and then constructed a cooperative module network (CMN). To analyze the functions and mechanisms of cooperative module pairs, we used the GO Term Finder to identify statistical significant enriched GO terms of a module (*p*-value < 0.01) as its annotation [39] (see Additional file 1 for details) and identified cell cycle-related genes in each module using the cell cycle-related gene set. The GO term with the most significant *p*-value was chosen as the function of a module (see Additional file 3). The cell cycle-related gene set contains genes that are cell cycle-regulated or whose functions are annotated as cell cycle or DNA processing in MIPS [12,40] (see the Methods section for details; genes are listed in Additional file 4). Genes contained in a module and in the cell cycle-related gene set were identified as cell cycle-related genes of the module (see Additional file 5). Information about modules that link more than three modules in the cooperative module network is listed in Table 1. For each of these modules, Table 1 presents its function, a subset of cell cycle-related genes contained in it, and the number of genes in it.

**Table 1:** Modules in the cooperative module network (CMN).

ID	No. genes^1^	Deg^2^	Function (p-value)^3^	Cell cycle-related genes^4^
0	91	34	response to DNA damage stimulus (3.93e-33)	*RAD24,RAD53,RAD9,DUN1,MEC1, MAD3,MRE11, MEC3,SGS1,CLB2*

2	59	13	Golgi vesicle transport (5.21e-12)	*CDC50,GCS1,RGP1,SEC28,KAR2*

30	27	13	cell morphogenesis (3.53e-09)	*CDC28,CLN4,PHO85,PCL2,SWE1, SWI4,FUS1,CDC12*

3	42	8	mitosis (4.92e-14)	*BUB3,MAD1,MAD2,MAD3,BFA1, RAD53,CLN3*

13	17	6	nucleosome assembly (1.99e-18)	*ABF1,ABF2,HTB1,HTB2,HHT1*

39	13	6	protein amino acid acetylation (9.87e-12)	*GCN5,TAF1,TRA1,SPT8*

22	15	5	nucleosome disassembly (4.47e-40)	*RCS1,RCS2,RCS4,RCS6,RCS8*

28	24	5	lipid biosynthetic process (6.24e-09)	*LAC1,GAS3*

4	27	4	ubiquitin-dependent protein catabolism (2.20e-30)	*RAD23,RPT1,RPT6,RPN11,RPN12*

14	5	4	regulation of transcription (G2/M-phase) (1.83e-08)	*FKH1,FKH2,NDD1,ACE2,SWI5*

32	11	4	regulation of cell division (1.55e-11)	*CDC28,CLN2,CLN3,STB1,FUS3*

54	7	4	protein folding (0.00015)	*HAP1*

^1^The number of genes contained in a module.

^2^The number of links of a module in the cooperative module network (CMN).

^3^For each module, we only listed the most significantly enriched functional GO categories (with *p*-value) (complete results are listed in Additional file 3).

^4^A subset of cell cycle-related genes contained in each module is listed in the last column (complete results are listed in Additional file 5).

Table 1 lists all modules that cooperate with more than four modules. For each module, we listed the most significantly enriched functional GO category (with *p*-value). We found that all of these modules were annotated to cell cycle-associated or specific functions. To further analyze specific functions and interactions mediated by each module, cell cycle-related genes reported in previous studies [40] were identified in each module (complete results are listed in Additional file 5). These genes include cell cycle-regulated genes and genes annotated in the functional categories of cell cycle and DNA processing. A subset of cell cycle-related genes contained in each module is listed in the last column. Cell cycle-related genes in each module that control the progression of the cell cycle, such as *CDC28*, cyclins, transcription factors, and checkpoint-related genes, are listed in the last column.

Furthermore, we measured the significance of gene correlations within and between the putative cooperative module pairs (see the Methods section for details). Two types of correlation, physical interaction and co-expression, were tested (see Additional file 6 for results). Our results show that correlations within all the 82 modules were significant and genes of each module are highly connected by physical interactions. Highly significant crosstalk relationships were also shown in our 50 predicted cooperative module pairs, indicating their pivotal roles in communication among biological pathways. By comparing the number of co-expressed gene pairs in the cell cycle with that in randomized expression datasets, we found that 67 out of 101 module pairs contain significant number of correlations between modules. Moreover, 31 out of 82 modules showed the significant number of correlations within modules. These results suggest that most of the identified module pairs (83 out of 101 module pairs) are significantly correlated.

As shown, most modules, especially those in Table 1 (purple circles in Figure 4), were annotated as cell cycle-related or other specific functions with statistical significance (*p*-values < 0.01). Essential regulators that control the progress of the cell cycle, such as *CDC28*, cyclins, transcription factors, and checkpoint-related genes, were also identified in functionally corresponding modules. The main cooperative relationships among modules and the basic function of and implicit crosstalk interactions between modules in the cell cycle are illustrated in Figure 4 and Table 1. For example, we found that 57 of 82 modules contain target genes of Cdc28 [41,42]. These results provide evidence for potential cooperative interactions between modules containing *CDC28 *and other modules. The importance of these modules can be explicitly demonstrated by the genes contained in them and the interactions in the cooperative module network (Figure 4 and Table 1). For example, module 0 (response to DNA damage stimulus) contains genes whose products sense DNA damage, activate the DNA repair system and pass this signal to other functional components such as modules involved in DNA replication [e.g., module 10 (maintenance of fidelity during DNA-dependent DNA replication), and module 12 (DNA replication initiation)] to induce appropriate cell responses. More results about cooperative interactions through essential regulators are discussed in Additional file 1.

#### Communication mechanisms and functions of phase-related cooperative modules

**Figure 5:**
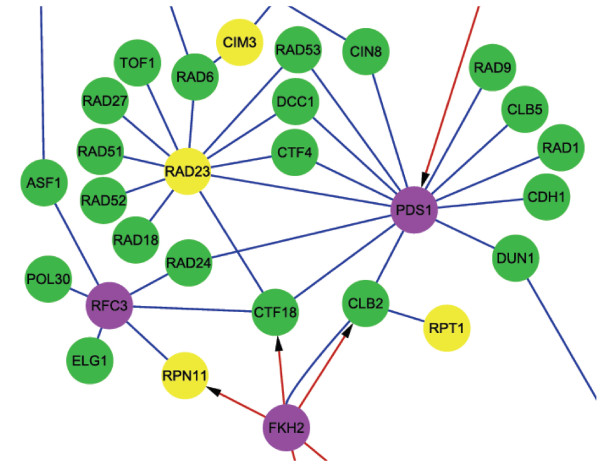
**Cooperating interactions related to the cooperative pair of module 0 and module 4**. This graph shows some of the interactions and crosstalk for module 0 (response to DNA damage stimulus) and module 4 (ubiquitin-dependent protein catabolism). Green circles indicate genes in module 0, and yellow circles indicate genes in module 4. Correlated genes of the cooperative module pair are represented by purple circles. Links represent regulatory interactions (red) and protein-protein interactions (blue) between two genes in the weighted physical interaction network (WPI network). Only crosstalk interactions and interactions with correlated genes are shown. *RAD23 *(YEL037C) is contained in module 4, and *FKH2 *(YIL131C) is a correlated gene of the module pair. The genes in module 4, particularly *RAD23*, are shown to interact directly with Module 0 and members of the 26S proteasome (contained in Module 4). Fkh1 regulates both module 0 and 4. By tracing crosstalk interactions between two modules and interactions with correlated genes, we can infer potential cooperating interactions of a module pair.

In combination with the interactions represented in the WPI network and identified correlated genes of each identified module pair (see Additional file 7), we then reconstructed a global map of the cooperative architecture of module pairs. Using module 0 (response to DNA damage stimulus) and module 4 (ubiquitin-dependent protein catabolism) as examples, possible interfaces of the two modules of an identified module pair and communication with other modules could be inferred by determining the direct physical interactions between the correlated genes and genes within the modules in the WPI network (Figure 5). The proteins in module 4 (ubiquitin-dependent protein catabolism), particularly Rad23 (YEL037C), are shown to interact directly with Module 0 (response to DNA damage stimulus) and members of the 26S proteasome. These connections suggest that Rad23 and Rad23-related ubiquitin/proteasome processes are all necessary for nucleotide excision repair and DNA damage checkpoints. In addition, Fkh1 (YIL131C) regulates both the DNA damage response and ubiquitin-dependent modules, suggesting that the function of these two modules might also be important in the G2/M phases of the cell cycle. These cooperative associations were also demonstrated in previous studies [43,44].

One module may cooperate with different modules and genes to promote progression through each phase of the yeast cell cycle. We performed hypergeometric tests to evaluate our results and to investigate cooperative module pairs that significantly associate with cell cycle progression (see the Methods section for additional details). Based on the tests, we found that 78 identified module pairs significantly associate with the cell cycle process and 67 identified module pairs significantly correlate with genes that are regulated in a specific phase of the cell cycle (complete results in Additional file 8). The number of cooperative module pairs that significantly correlate with genes that are functional in early G1, late G1, S, G2, and M phase are 14, 36, 18, 15, and 20, respectively (complete results in Additional file 8). To discover the most essential cooperating module pairs and to determine their functions, we ranked the 67 phase-related module pairs according to the number of their correlated genes that are regulated in a specific cell cycle phase. Genes regulated in a specific phase were identified with the phase-regulated gene set from Cho *et al. *[45] (see the Methods section for additional details; genes are listed in Additional file 9). In this way, the top three module pairs were chosen for each phase of the cell cycle (Table 2). To analyze the mechanisms of the 15 phase-related cooperative associations, we collected correlated genes of each module pair that are regulated in the corresponding phase of cell cycle and genes in the module pair that are connected with these correlated genes by direct physical interactions. In addition, we also collected genes that mediate crosstalk links (direct physical interactions) between two modules of a module pair.

**Table 2:** Phase-related cooperative module pairs (the top three).

Phase	Pair_ID^1^ (*p*-value)	Genes (1^st^)	Genes (2^nd^)	CorrGenes^2^
Early G1 (3, 4)	93(30,32) (0.000168)	*SWI4*, *CDC28*, *BNI1*, *FUS1*	*FUS3, STE5, CCD28, CLN2, CLN3*	*SWI4, FAR1*

(5, 6)	50(42,30) (0.00174)	*CDC28, CDC34, CDC4, CLN2*	*CDC28, BNI1, SLT2, PKC1, SWE1*	*CDC6, FAR1*

(7, 8)	37(32,0) (0.0066)	*CDC28, CLN2, CLN3, STB1*	*RAD9, RAD53, SWI6, MRC1, MEC1, CDC45*	*SWI4*

Late G1 (9, 10, 11)	13(0,30) (6.68E-11)	*RAD18, RAD6, RAD53, CLB5*	*SWE1, CDC28, SWI4*	*HO*, *CLB6*, *SWE1*

(12, 13)	20(0,15) (0)	*RAD53, RAD52*,	*RAD1*, *RAD14*	*CDC9*

(14, 15, 16)	15(0,10) (0)	*POL30,POL32, RAD9*	*MSH2, MSH3, MSH6, MLH1, EXO1*	*POL30, MSH6, MSH2*

S (17, 18)	2(3,0) (7.52E-09)	*CIN8, KIP3, MAD2*	*RAD53, DUN1, MEC1, CDH1*	*CIN8,PDS1*

(19, 20)	43(37,17) (4.07E-08)	*SPC24,DAM1, CBF2*	*MTW1, NNF1, NSL1, DSN1, NDC80*	*MTW1,SPC25,SPC110, CBF2*

(21, 22)	34(3,27) (1.71E-06)	*BIM1, CIN8, KAR9*	*BIK1*	*CIN8, BIM1*

G2 (23)	1(1,0) (0.00027)	*MOB1, SWI4, SIC1, CDC28*	*DBF2, LTE1, CLB2, SRS2*	*MOB1, SRS2, DBF20*

(24, 25, 26)	5(39,0) (0.00015)	*GCN5, SPT8*	*RAD51, RAD53, ASF1, RAD6, MEC1*	*HTZ1*

(27, 28)	50(42,30) (0.0029)	*CDC34, CDC4, CDC28*	*SWE1, PKC1*	*ELM1, CHS2*

M (29, 30)	47(30,14) (2.3E-08)	*PCL2, PHO85, CDC28*	*SWI5, ACE2, FKH2, FKH1, NDD1*	*CLB3, SWI5, ACE2, CDC5, BUD4, CDC28*

(31, 32)	92(3,79) (2.95E-08)	*BUB3, CLB3, CLB5*	*SIC1, CLB5, CDC28*	*CLB3, CLB1, CLB4*, *CDC20*

(33, 34, 35)	93(30,32) (8.91E-05)	*SLT2,CDC12, CDC28*	*CLN1, CLN2, FUS3, CDC28*	*MSG5, BUD2*

^1^**Pair_ID **indicates the unique identifier of each identified module pair; (ID_1, ID_2): indicates the unique identifiers (IDs) of the first and second modules of a module pair, respectively.

^2^**CorrGenes **indicates correlated genes of a module pairs that are regulated in the corresponding phase.

To discover cooperative module pairs essential for controlling cell cycle progression, we ranked the 67 phase-related module pairs (complete results in Additional file 8) according to the number of phase-regulated genes correlated with each module pair and then listed the top three module pairs for each phase. The statistical significance of the cooperation of a module pair (***p*-value**) and the ID of a module pair are listed in the second column. To assess the effects and mechanisms of cooperation, a subset of regulated correlated genes for each module pair and genes demonstrated to be associated with these correlated genes are also listed in the table. These genes include genes contained in the first module (**Genes (1^st^)**) and in the second module (**Genes (2^nd^)**) of a module pair. The references for these cooperative interactions are listed in Additional file 1 Table A1. The serial numbers of these references are shown in the first column.

As shown in Table 2, these cooperative associations between cell cycle-specific components were verified in previous studies. We also found that the interactions among these modules and their phase-regulated correlated genes occur mostly through regulators that control the cell cycle and the transcription of phase-regulated genes and checkpoint-related genes. As shown in our results, cell cycle phase transitions are accompanied by changes in the main functional modules and their interactions (see Additional file 1 for more discussions). Cooperative relationships of modules in each phase of the cell cycle seem to be established differently by different gene interactions within modules. For example, genes that function in response to DNA damage stimulus (genes in module 0) can communicate with genes related to mismatch repair (genes in module 10) via Pol32 (late G1 phase in Table 2) or with mitosis-related genes (genes in module 3) at the S and G2/M checkpoints via Rad53 (S and G2 phase in Table 2). Moreover, we found that signal transduction among modules occurs mainly through Cdc28 and that Cdc28 associates with different cyclins, transcription factors and genes regulated in different phases to promote cell cycle progression (early G1, late G1, G2, and M phase in Table 2). Thus, we believe that these cooperative associations cover important operations in each phase.

#### Module crosstalk networks under the regulation of Cdc28, phase-related cyclins, cell division cycle genes (CDC genes) and transcription factors

**Figure 6:**
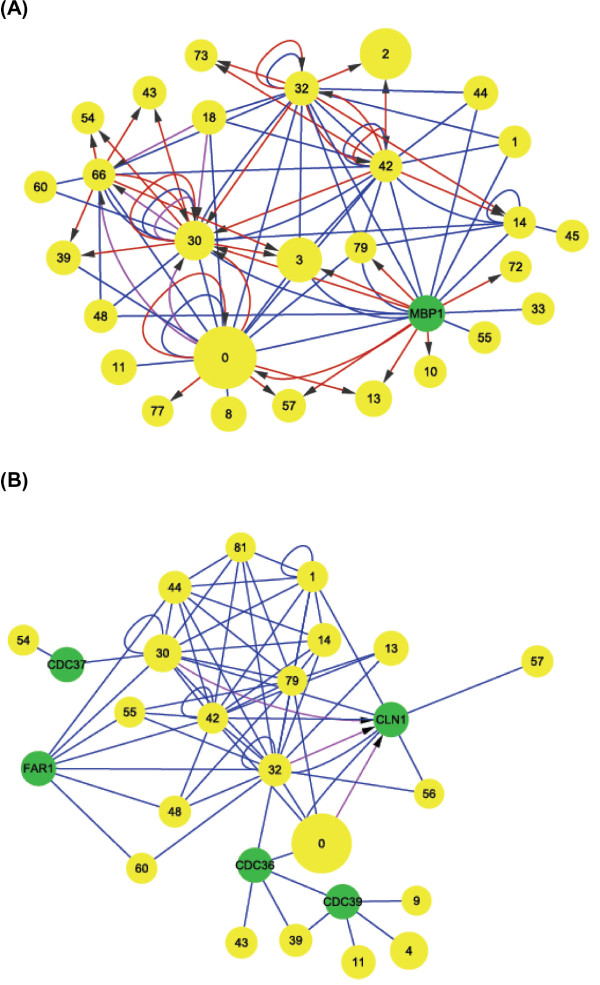
**Crosstalk relationship graphs of G1 phase**. We constructed relationship graphs based on significant crosstalk interactions through G1-specific regulators (see the Methods section for details). Yellow circles indicate modules from identified cooperative module pairs, and green circles indicate regulators not contained in any modules. The size of the yellow circles is proportional to the number of genes within the modules. Links indicate either relationships dependent on a significant number of protein-protein interactions (blue links) or relationships dependent on a significant number of regulatory interactions (red or purple links) (see the Methods section for details). Only relationships among correlated genes and their correlated module pairs were used to construct the relationship graphs (Figure 3). When a phase-specific transcription factor was contained in module *X *and regulated a significant number of genes in module *Y*, a red link was drawn from the circle representing *X *to the circle representing *Y*. On the contrary, if a phase specific-regulator was regulated by more than one factor in module *Z*, a purple link was drawn from the circle representing *Z *to the circle representing the module containing the regulator or the circle indicating the regulator. (A) Relationship graph of G1-related transcription factors. Factors considered in the graph include *SWI4*, *MBP1*, *SWI6*, *STB1*, *ACE2*, and *SKN7*. *SWI4 *is contained in module 30 (cell morphogenesis) and module 66 (amino sugar metabolic process). *SWI6 *and *SKN7 *are contained in module 0 (response to DNA damage stimulus). *STB1 *is contained in module 32 (regulation of cell division) and module 42 (G1/S transition of mitosis and interphase). *ACE2 *is contained in module 14 (regulation of transcription during G2/M phase interphase). (B) Relationship graph of *CDC28*, G1-related cyclins and CDC genes. In this graph, we focused on module crosstalk through Cdc28, Cln1, Cln2, Cln3, Cdc36, Cdc37, Cdc39, Far1 and Fus3. *CDC28 *is contained in module 1 (regulation of cell cycle), module 30 (cell morphogenesis), module 32 (regulation of cell division), module 42 (G1/S transition of mitotic cell cycle and interphase), module 44 (regulation of mitosis), and module 79 (regulation of cyclin-dependent protein kinase activity). *CLN2 *is contained in module 32 (regulation of cell division), module 42 (G1/S transition of mitotic cell cycle and interphase), and module 79. *CLN3 *is contained in module 32. *FUS3 *is contained in module 32.

**Figure 7:**
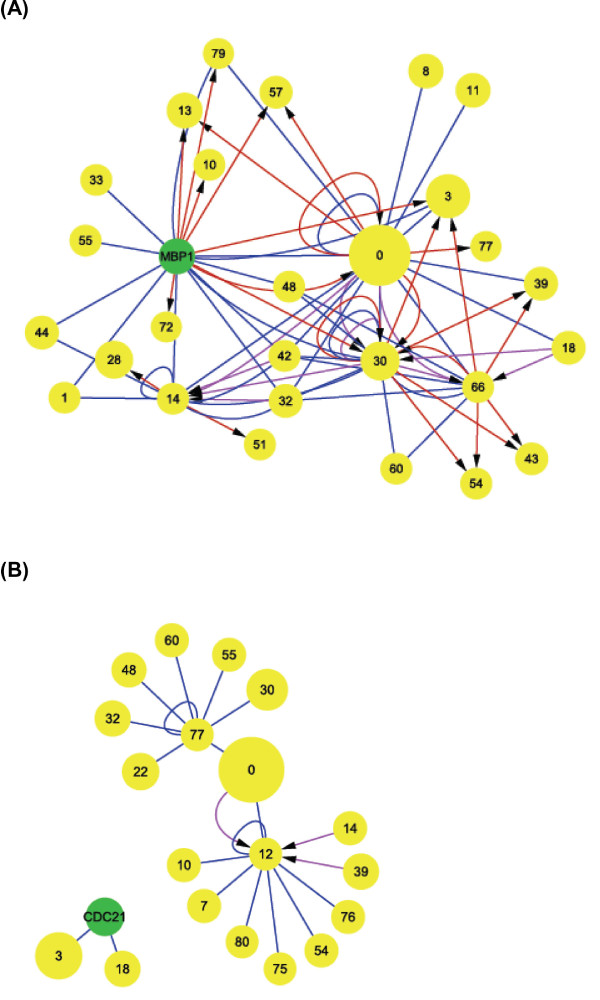
**Crosstalk relationship graphs of S phase**. Yellow circles indicate modules from identified cooperative module pairs, and green circles indicate regulators not contained in any modules. The size of the yellow circles is proportional to the number of genes within the modules. Links indicate either relationships dependent on a significant number of protein-protein interactions (blue links) or relationships dependent on a significant number of regulatory interactions (red or purple links) (see the Methods section for details). Only relationships among correlated genes and their correlated module pairs were used to construct the relationship graphs (Figure 3). When a phase-specific transcription factor was contained in module *X *and regulated a significant number of genes in module *Y*, a red link was drawn from the circle representing *X *to the circle representing *Y*. On the contrary, if a phase specific-regulator was regulated by more than one factor in module *Z*, a purple link was drawn from the circle representing *Z *to the circle representing the module containing the regulator or the circle indicating the regulator. (a) Relationship graph of S phase-related transcription factors. Factors considered in the graph include *SWI4*, *SWI6*, *MBP1*, *NDD1*, and *Skn7*. *SWI4 *is contained in module 30 (cell morphogenesis) and module 66 (amino sugar metabolic processes). *SWI6 *and *SKN7 *are contained in module 0 (response to DNA damage stimulus). NDD1 is contained in module14 (regulation of transcription during G2/M phase interphase). (b) Relationship graph of S phase-related CDC genes. Cdc7, Cdc8, Cdc21, and Cdc24 are considered in the graph. *CDC7 *is contained in module 12 (DNA-dependent DNA replication initiation). *CDC24 *is contained in module 77 (regulation of nuclear division).

**Figure 8:**
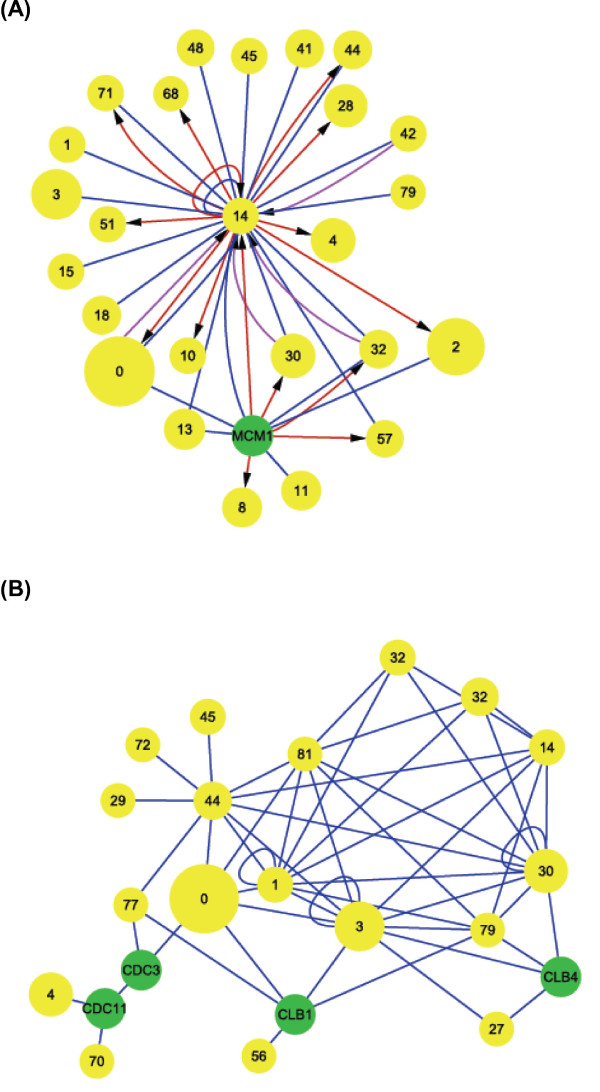
**Crosstalk relationship graphs of G2 phase**. Yellow circles indicate modules from identified cooperative module pairs, and green circles indicate regulators not contained in any modules. The size of the yellow circles is proportional to the number of genes within the modules. Links indicate either relationships dependent on a significant number of protein-protein interactions (blue links) or relationships dependent on a significant number of regulatory interactions (red or purple links) (see the Methods section for details). Only relationships among correlated genes and their correlated module pairs were used to construct the relationship graphs (Figure 3). When a phase-specific transcription factor was contained in module *X *and regulated a significant number of genes in module *Y*, a red link was drawn from the circle representing *X *to the circle representing *Y*. On the contrary, if a phase specific-regulator was regulated by more than one factor in module *Z*, a purple link was drawn from the circle representing *Z *to the circle representing the module containing the regulator or the circle indicating the regulator. (a) Relationship graph of G2-related transcription factors. Factors considered in the graph include Fkh2, Fkh1, Ndd1, and Mcm1. These factors are all contained in module 14 (regulation of transcription during G2/M phase interphase). (b) Relationship graph of G2 phase-related CDC genes, cyclins, and CDC28. Ccd3, Ccd5, Ccd11, Ccd14, Ccd15, Ccd28, Clb1, Clb2, Clb3, and Clb4 are considered in the graph. *CDC5*, *CDC14*, and *CDC15 *are contained in module 44 (regulation of mitosis). *CDC28 *is contained in module 1 (regulation of cell cycle), module 30 (cell morphogenesis), module 32 (regulation of cell division), module 42 (G1/S transition of mitotic cell cycle and interphase), module 44 (regulation of mitosis), and module 79 (regulation of cyclin-dependent protein kinase activity). *CLB2 *is contained in module 0 (response to DNA damage stimulus). *CLB3 *is contained in module 3 (mitosis).

**Figure 9:**
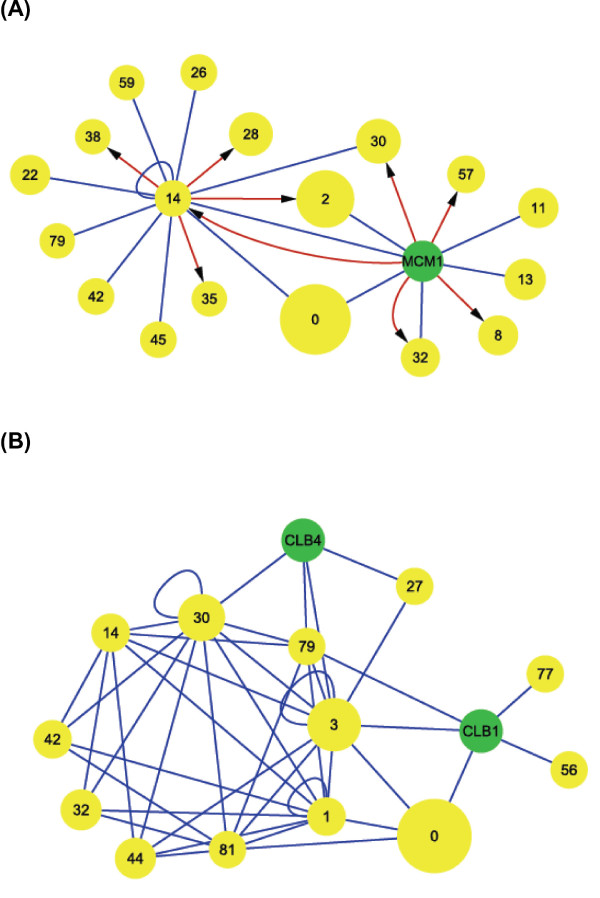
**Crosstalk relationship graphs of M phase**. Yellow circles indicate modules from identified cooperative module pairs, and green circles indicate regulators not contained in any modules. The size of the yellow circles is proportional to the number of genes within the modules. Links indicate either relationships dependent on a significant number of protein-protein interactions (blue links) or relationships dependent on a significant number of regulatory interactions (red or purple links) (see the Methods section for details). Only relationships among correlated genes and their correlated module pairs were used to construct the relationship graphs (Figure 3). When a phase-specific transcription factor was contained in module *X *and regulated a significant number of genes in module *Y*, a red link was drawn from the circle representing *X *to the circle representing *Y*. On the contrary, if a phase specific-regulator was regulated by more than one factor in module *Z*, a purple link was drawn from the circle representing *Z *to the circle representing the module containing the regulator or the circle indicating the regulator. (a) Relationship graph of M phase-related transcription factors. Factors considered in the graph include Ace2, Swi5, and Mcm1. *ACE2 *and *SWI5 *are contained in module 14 (regulation of transcription during G2/M phase interphase). (b) The relationship graph of G2 phase-related CDC genes, cyclins, and *CDC28*. Ccd28, Clb1, Clb2, Clb3, and Clb4 are considered in the graph. *CDC28 *is contained in module 1 (regulation of cell cycle), module 30 (cell morphogenesis), module 32 (regulation of cell division), module 42 (G1/S transition of mitotic cell cycle and interphase), module 44 (regulation of mitosis), and module 79 (regulation of cyclin-dependent protein kinase activity). *CLB2 *is contained in module 0 (response to DNA damage stimuli). *CLB3 *is contained in module 3 (mitosis).

**Figure 10:**
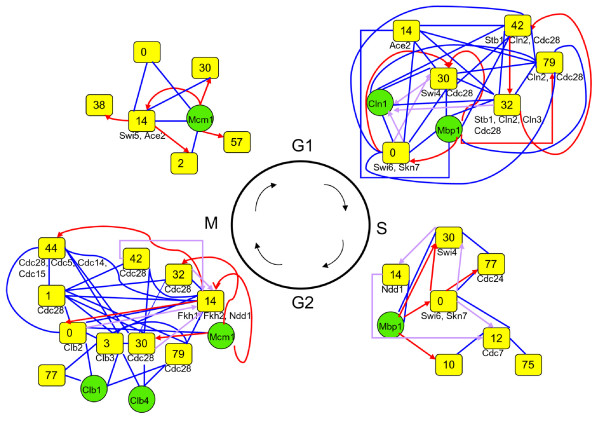
**Summary of crosstalk relationship graphs for the yeast cell cycle**. Crosstalk relationship graphs (Figure 6, 7, 8 and 9) belonging to the same phase were combined to form one graph. The associations mediated by Cdc28, cyclins, and CDC genes during G2 and M phase were merged into the graph of G2 phase. Only the most essential and specific associations of each crosstalk relationship graph that were demonstrated are shown. The references for these crosstalk associations are listed in Additional file 1 Table A2. The center of the summary graph shows each phase of the cell cycle. Each graph is placed according to the related stage of the cell cycle. In the graph, yellow boxes labeled "module ID" indicate modules, and green circles indicate regulators not contained in any modules. Phase-related regulators contained in a module are listed under the circle presenting the module. Links indicate either relationships dependent on protein-protein interactions (blue links) or relationships dependent on transcriptional regulation (red or purple links). Only relationships among correlated genes and their correlated module pairs were used to construct the relationship graphs (see the Methods section for details).

**Figure 11:**
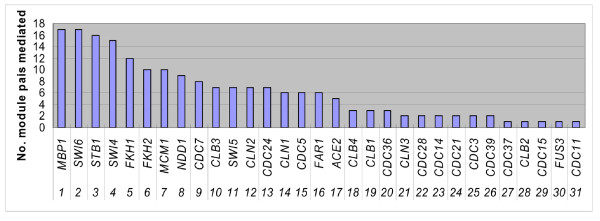
**Number of cooperative relationships mediated by phase-specific regulators**. For each phase-specific regulator listed in Table 3, the number of cooperative module pairs that correlate with the regulator is shown in Figure 11.

Although previous studies have focused on functions of the essential cyclin-dependent kinase Cdc28, cell division cycle genes (CDC genes) and related transcription factors, the crosstalk between modules controlled by Cdc28 and phase-specific regulators is still not clear. We analyzed the cooperative relationships (Figure 3) of the correlated genes in each cooperative module pair to identify direct crosstalks that involve the regulation of Cdc28, known phase-related CDC genes, cyclins or transcription factors [2] (see the Methods section for additional details). Table 3 lists the regulators for each phase and modules containing these regulators. Finally, we constructed crosstalk relationship graphs for each cell cycle phase. Figures 6A, 7A, 8A, and 9A show interacting relationships between modules controlled by specific transcription factors in G1, S, G2 and M phases. Figures 6B, 7B, 8B, and 9B show the CDC genes, cyclins and Cdc28-associated crosstalk relationships of G1, S, G2 and M phases. In these graphs, module pairs mediated by these regulators and modules that contain these regulators were investigated (detailed information is in Additional file 10 and Additional file 11). The essential and specific associations of each relationship graph that were previously reported are summarized in Figure 10. The associations mediated by Cdc28, cyclins, and CDC genes during G2 and M phases were merged into the graph of G2 phase in Figure 10. Thus, by identifying phase-specific module interactions involved in cell cycle regulation, we can further determine the influences and functions of module interactions and regulators in controlling the cell cycle (see Figure 10). A total of 59 module pairs that correlate with these regulators were identified (see Additional file 10). Figure 11 shows the number of identified module pairs mediated by each regulator.

**Table 3:** Regulators of each phase.

Phase	Transcriptional Factors	CDC28/cyclins/CDC genes	Modules containing these factors (ID)
G1	*SWI4, MBP1, STB1, SWI6, ACE2, SKN7*	*CLN3, FUS3, FAR1, CDC36, CDC39, CLN2, CDC37, CDC28, CLN1*	0, 1, 14, 30, 32, 42, 44, 66, 79

S	*SWI4, MBP1, NDD1, SWI6, SKN7*	*CDC24, CDC7, CDC8, CDC21*	0, 12, 14, 30, 66, 77

G2	*FKH1, FKH2, NDD1, MCM1*	*CDC11, CLB2, CDC15, CLB4, CDC28, CDC3, CDC5, CDC14, CLB1, CLB3*	1, 3, 14, 30, 32, 42, 44, 79

M	*MCM1, SWI5, ACE2*	*CDC28, CLB1, CLB2, CLB3, CLB4*	0, 1, 3, 14, 30, 32, 42, 44, 79,

Essential regulators, including Cdc28, cyclins, CDC genes, and phase-specific transcription factors are listed for each phase. Relationship graphs of each phase were constructed via cooperative interactions through regulators of the phase (Figure 6,7,8,9, and 10). To trace regulators that mediate crosstalk between cooperative modules, the IDs of modules that contain these regulators are also listed in the final column.

When only connections via protein-protein interactions were considered, the relationship graphs of Cdc28 and CDC genes of G1, G2, and M phase (Figure 6B, 8B9B and 10) and the relationship graph of G1 and G2 phase-related transcription factors showed a compact connectivity of modules (Figure 6A, 8A and 10). This type of connectivity implies that modules display direct crosstalk with each other. For example, modules 0 (response to DNA damage stimulus), 32 (regulation of cell division), and 42 (G1/S transition of mitotic cell cycle and interphase) all contain Cdc28, and these modules connect to each other to form a clique-like subgraph in the relationship graph of Cdc28, cyclins, and CDC genes (Figure 6B and G1 phase of Figure 10). These relationships are primarily due to interactions involving Cdc28, related-cyclins, Cdc28 substrates and phase-related transcriptional factors. Regulatory relationships of S, G2 and M phase are mainly mediated by module 14 (regulation of transcription during G2/M phase) (Figure 7A, 8A, 9A and 10). Transcriptional factors essential in progression of S to M phase are contained in module 14. These relationships suggest possible cooperations among functional modules for regulating the progression of each phase (see Additional file 1 for more discussions of each relationship graph and references).

## Discussion

To construct a global map of cooperative functional components in a specific cellular process, we developed an approach to gather more information and to better understand interactions between different functional modules. As an example, we applied this approach to the yeast cell cycle. Using this methodology, we identified genes and interactions related to the regulation and signal transduction of cooperative functional components in the cell cycle, in addition to cooperative module pairs. The structures and properties of module cooperation in the cell cycle were also revealed by our analyses. Most of these results are consistent with previous studies and can be used to explain the complex operation of the cell cycle.

The weighted physical interaction network, search algorithm, and analytical methods enhanced the ability of our approach to identify condition-specific cooperative modules and to decipher mechanisms of module cooperation. We designed the weighted physical interaction network to capture dynamic information about genes and to measure the relationships between genes and modules. The weighted physical interaction network can be treated as a platform for integrating information from different types of experimental data. Thus, the correlations between modules identified by the spanning algorithm will not be restricted to only one type. Our methods can also identify important associations and genes related to module cooperation. In the cooperative module network analysis, we constructed an association graph of the cell cycle response to DNA damage stimulus using cooperative module pairs identified from the previous step. Based on functional annotation by GO and the cell cycle-related genes contained in each module, we were able to infer specific functions of the cooperative associations and the identified modules. Most modules were found to be essential for the cell cycle and important for module cooperation during different phases of the cell cycle. Examples include modules 0 (response to DNA damage stimulus), 30 (cell morphogenesis) and 3 (mitosis). Module 3 associated with other modules specific to mitosis, whereas module 0 (response to DNA damage stimulus) and module 30 (cell morphogenesis) associated with modules of more than one phase in the cell cycle (Figure 4, Figure 10, Table 2). These results highlight the important roles of these modules and the cooperative associations among them.

Based on our phase-related module pair analysis, we further inferred detailed interaction dynamics of each cooperative module pair during various phases of the cell cycle. For example, to initiate appropriate responses to DNA damage, module 0 cooperates with genes within specific modules in the G1, S and G2 phases (Table 2). Similarly, module 30 interacts with genes expressed in the G1, G2 and M phases to regulate cell morphogenesis. These interactions also suggest possible mediators of these associations and specific functions of them in the cell cycle. In this analysis, we also calculated the number of correlated genes regulated in a specific phase of the cell cycle and thereby ranked the importance of the module pairs to each phase. These results highlight the main interactions among functional components in each phase (Table 2).

Finally, relationship graph analysis was also performed to display crosstalk between identified modules. This analytical method was designed to identify crosstalk mediated by a set of regulators, Cdc28, cyclins, cell cycle division-related genes (CDC genes) and phase-related transcription factors. From the relationship graphs, we could easily visualize the most essential and direct regulatory interactions in the process and discover phase-specific regulation. For example, Cdc28 was strongly associated with the crosstalk among a group of functional modules related to mitosis and was correlated with transcriptional regulators such as Fkh1, Fkh2, and Ndd1 during G2/M phase (G2 phase in Figure 10) [42,46].

## Conclusions

Using the approach described here, we comprehensively identified dynamic assembling or activating modules and the cooperative relationships between them. Following several analytical steps, a map of dynamic cooperative associations was constructed by identifying regulators, regulated genes and interactions correlated with cooperative module pairs. This approach could be helpful in deciphering the cooperative mechanisms of a specific condition. The advantages of this methodology in identifying important components, interactions and genes in the yeast cell cycle were demonstrated by our results. Moreover, this approach can combine other data such as significantly regulated gene sets or known regulators to infer associations among functional components that are mediated by the gene sets and regulators. Thus, it could also be useful in predicting specific functions of assigned gene sets, modules or interactions. As our methodology is quite flexible, it could easily be applied to experimental data from different species, conditions, or biological techniques. Thus, by comparing results from different data sets, we should be able to identify unknown properties of dynamic cooperative interactions and gather new insights into dynamic cooperation mechanisms and condition-specific components.

## Methods

### Construction of a weighted physical interaction network (WPI network)

In the first step (Figure 1A), to predict probable functional correlations among genes in the cell cycle, we constructed a co-expression gene network based on gene expression profiles during the yeast cell cycle from Cho *et al. *[45] (obtained from ExpressDB [47]). A node of the co-expression network represents a gene, and a link (or edges) represents the significant expression correlation between two genes. Pairs of genes with Pearson correlation scores above 0.683 or below -0.683 were selected and considered to be significant positive and negative co-expression, respectively (see Additional file 1 for more details).

We then designed a weighted physical interaction (WPI) network using ChIP-chip data, protein-protein interaction data, and the co-expression network established in the previous step. The ChIP-chip data set was obtained from Harbison *et al. *[48]. Protein-protein interaction data identified with different experimental techniques for yeast were downloaded from the BioGRID database [13] and are shown in the Additional file 12. Nodes in the WPI network represented genes, links represented protein-protein interactions from BioGRID and regulatory relationships from ChIP-chip data. Based on these data, we generated an undirected link for each protein-protein interaction and directed links from transcription factors to target genes. Finally, the degree (the number of links) of each gene in the co-expression network was assigned to each corresponding gene and represents the weight of each gene in the WPI network.

### The consistency score

The consistency score (CT_score; Equation 1) measures the difference between the weights of genes correlated with a pair of modules and the weights of genes that are related to only one of the modules in the WPI network.(1)

In Equation 1, *G *is a gene set that consists of all genes in yeast; *NL_i _*is the number of physical links to gene *i *in the weighted physical interaction network; *N *is the total number of genes in yeast; *CopL_i _*is the weight of gene *i *in the WPI network; and *N_m1,i _*and *N_m2,i _*are the observed numbers of physical links connecting gene *i *and the genes in modules *m1 *and *m2*, respectively, in the WPI network. *M1 *and *M2 *are the numbers of genes contained in modules *m1 *and *m2*, respectively. (*M1*/N)*NL_i _and (*M2*/N)*NL_i _are used to estimate the expected number of links from genes in modules *m1 *and *m2*, respectively, to gene *i*.

### The mediation score

The mediation score (CoopMed; Equation 3) helped us to incorporate genes that mediate interactions among genes in the module but rarely link to genes outside of the module. The CMRatio score was used to measure the ratio of shared interacting partners of two genes.(2)(3)

The CMRatio*_i_*,*_j _*(Equation 2) is used to estimate whether gene *j *should be included in the module containing gene *i*. *CL_i,j _*is the number of genes linked by both genes *i *and *j *in the weighted physical interaction network. Thus, the more common neighbors between gene *i *and gene *j*, the greater the possibility that gene *i *and gene *j *are in the same module. Considering a pair of genes *s *and *t *as a seed (an initial module pair), a gene pair is a special case of a module pair (i.e., each module of a module pair contains only one gene). The CT_score*_i,seed _*is the consistency score of gene *i *and the initial gene in the other module. For example, when a seed comprising genes *s *and *t *is used to extend modules *m1 *and *m2*, respectively, the CT_score*_i,seed _*of gene *i *in *m1 *is the consistency scores of gene *i *and gene *t*. CoopMed_*j*,*m *_measures the consistency score of genes *j *and *t *when cooperating interactions of *j *and *t *are mediated by gene *i*. *R *is the probability that the link between gene *i *and gene *j *is real. *R *was set to 0.9 according the parameter *β *from a previous study [49].

### The cooperation score (MCoop_score)

The cooperation score (MCoop_score; Equation 4) was used to estimate the essentiality of the correlation of a module pairs and a scoring function of spanning algorithm. Consider a seed: *m1*={*s*} and *m2*={*t*} and a gene *u *contained in the same module with *s*. The CT_score*_u,seed _*is the consistency score of gene *u *and the initial gene *t *(Equation 3 in the Results section). CT_score___P*_u _*is the consistency score of gene *i *that maximizes the mediation score of *u *and an initial gene (*s *or *t*) of the other module (CoopMed*_u,m1_*).(4)

### The procedure for identifying cooperative module pairs

1. Construct a WPI network. (Figure 1A).

2. Select gene pairs with significantly high consistency scores to be initial module pairs (seeds) (step 2 in Figure 2; see Additional file 1 for more details). A gene pair is a special case of a module pair (i.e., each module of a module pair contains only one gene). Hence, we can calculate the consistency scores (CT_core; Equation 1) of all (N*(*N*-1))/2 gene pairs. Rank the non-zero consistency scores in descending order and select gene pairs with consistency scores above the 99^th ^percentile as seeds.

3. For each seed, apply our spanning algorithm to extend a module pair that maximizes the cooperation score (MCoop_score of the module pair; Equation 4 and step 3 in Figure 2).

4. Iteratively merge highly overlapped modules until no more modules can be merged. Consider two modules. If more than two-thirds genes of one module are also contained in the other module, the two modules are treated as highly overlapped modules. We treated highly overlapped modules as modules with identical functions.

5. Remove module pairs that are comprised of highly overlapped modules (step 5 in Figure 2). Cooperative correlations between overlapped modules are regarded as correlations within the same module. A module pair that consists of highly overlapped modules will be removed.

### The spanning algorithm

The spanning algorithm was used to extend a seed (an initial module pair) to a pair of modules that maximize the cooperation score (MCoop_score; Equation 4 in Methods). Consider a seed contains gene s and gene t: *m1*={*s*} and *m2*={*t*}. The spanning algorithm searched a pair of sub-networks in the weighted physical interaction network (WPI network) that maximize the cooperation score (MCoop_score) and contain *s *and *t*, respectively. Genes of each sub-network were assigned to the corresponding modules.

### Pseudo-code of the spanning algorithm

Main_Function:

Input:

A weighted physical interaction network (WPI network)

A seed: an initial module pair *m1*={*s*} and *m2*={*t*}

1. Construct a gene set *N *by adding all genes in the WPI network to *N*

2. Max_score_*m1m2*= *CT_score_s_*,*_t_*

3. call Sub Function: Module_extend(the WPI network, gene *t*, a module pair: *m1 *and *m2*, Max_score_*m1m2*, gene *s*, *N*, *R *= 0.9)

4. call Sub Function: Module_extend(the WPI network, gene *s*, module pair: *m2 *and *m1*, Max_score_*m1m2*, gene *t*, *N*, *R *= 0.9)

#*R *was set to 0.9 according the parameter *β *from a previous study [49].

Sub Function: Module_extend

Input:

The weighted physical interaction network (WPI network)

Cooper_center: a gene *t*

A module pair *M1 *and *M2*

Max_score

Initial gene: a gene *y*

Visit_list: a gene set *N*

*R *# *R *is the probability that the physical interaction between a gene pair is real.

1 If (there is a neighbour of *y *is contained in *N*)

2    select the gene *i *that is a neighbour of *y *with the largest *CMRatiy_y_*,*_i _*from *N*

3    remove *i *from *N*

4    add *i *to *M1 *and count MCoop_score_*M1*,*M2*_

5    If (MCoop_score_*M1*,*M2 *_>= Max_score)

6       Max_score = MCoop_score_*M1*,*M2*_

7       If (*CT_score_i_*,*_t _*>= *CT_score_y_*,*_t_*)

8          call Sub Function: Module_extend(the WPI network, gene *t*, *M1 *and *M2*, Max_score, gene *i*, *N*, *R *= 0.9)

9 Else

10   remove *i *from *M1 *and return

### Evaluation of correlations within and between modules

To evaluate the correlations within and between modules of an identified module pair, we measured the significance of gene correlations within each module and between modules. We tested two types of correlation: physical interaction and co-expression. The significance of physical interaction within and between modules is measured by comparing the number of physical interactions within and between modules found in the WPI network to that found in random networks (see Additional file 1 for details). Similar methods to measure the correlations of gene expression patterns had been previously proposed [50,51]. To measure the significance of co-expressed correlations within and between modules of each identified module pair, we compared the number of co-expressed gene pairs within and between modules found in the cell cycle expression dataset from Cho *et al. *[45] with that found in randomized expression datasets (see Additional file 1 for details).

### Datasets used in this study

To evaluate our method, we used a cell cycle-related gene set and a phase-regulated gene set. The cell cycle-related gene set consisted of 985 genes from three types of benchmark sets, including genes significantly regulated in the cell cycle and genes annotated in functional categories of cell cycle and DNA processing [40] (genes are listed in Additional file 4). The phase-regulated gene set consisted of 416 genes with significant periodically changing expression identified by Cho *et al. *[45] and was divided into five groups: genes regulated in early G1 phase, late G1 phase, S phase, G2 phase, and M phase (genes are listed in Additional file 9).

### Statistical evaluation of the cooperation of identified module pairs

We assessed the significance of the cooperation of a module pair in a specific phase of the cell cycle using a hypergeometric test.(5)

where *G *is the number of genes in the yeast genome; *C *is the number of correlated genes of the cooperative module pair; *b *is the number of correlated genes of the module pair that are also in the previously reported gene set *D*; *B *is the number of genes in *D*. We estimated the statistical significance of the association of a phase with the correlated genes of a module pair. For estimating the statistical significance of the association of a phase, *D *was assigned the phase-regulated gene set that consists of genes regulated in a specific phase (G1/S/G2/M) reported by Cho *et al. *[45] (genes are listed in Additional file 9). Otherwise, *D *was assigned the cell cycle-related gene set (genes are listed in Additional file 4) to identify module pairs that significantly associate with the cell cycle process. The significance of the cooperation of a module pair in the cell cycle process was also evaluated. Module pairs with *p*-values < 0.05 were considered significant in the cell cycle process or phase.

### Construction of the relationship graph for each phase of cell cycle

To present a map of cooperative regulation and interactive mechanisms between identified modules in the cell cycle, we constructed relationship graphs for each phase by combining correlated genes of module pairs (genes are listed in Additional file 7) and phase-related regulators (see Table 3). In these relationship graphs, each identified module and regulators not located in any of these modules are represented by a node. To present significant associations, we only consider regulators that are correlated genes of a module pair and relationships among correlated genes and their correlated module pairs (Figure 3). In each relationship graph, a link between two modules can be treated as a significant crosstalk relationship mediated by regulators between either two different modules or a module and a regulator that are not located in any module (see Additional file 1 for more details). Directed links represent transcriptional associations and undirected links represent protein-protein associations. Consider a cooperative module pair, module *m1 *and module *m2*, and the regulator *x *(one of the regulators listed in Table 3). If *x *is a correlated gene of the module pair that regulates (by either transcriptional or protein-protein interactions) or is regulated by a significant number of genes in module *m2*, a link will be generated between either the module containing *x *or *x *and module *m2 *according to the type of interaction. Similarly, a link will be generated between either the module containing *x *or *x *and module *m1*. For example, if *x *is contained in *m1 *and is transcriptionally regulated by a significant number of genes in *m2*, a directed link will be generated from *m2 *to *m1*. Finally, we excluded modules without a link from each relationship graph.

## Competing interests

The authors declare that they have no competing interests.

## Authors' contributions

JTH developed the method, performed the analyses and wrote the manuscript. CHP performed the data analyses and wrote the manuscript. WPH, CYL, and CYT advised on method design and wrote the manuscript. CYT investigated the principle. All authors read and approved the final manuscript.

## Supplementary Material

Additional file 1 **Supplementary discussions**. Additional details of our method and discussions were described in Additional file 1. Click here for file

Additional file 2 **Gene lists of the identified modules**. Additional file 2 lists the genes in each identified module. Click here for file

Additional file 3 **Functional annotation results o f the identified modules**. Additional file 3 lists functional annotation results of the 82 modules. We annotated functions of the identified modules from biological processes of Gene Ontology and listed the most significant function of each module. Click here for file

Additional file 4 **Cell cycle-related gene set**. The cell cycle-related gene set consisted of 985 genes from three types of benchmark sets, including genes significantly regulated in the cell cycle and genes annotated in functional categories of cell cycle and DNA processing [40]. Click here for file

Additional file 5 **Cell cycle-related genes of the identified modules**. Genes that are cell cycle-regulated and/or functional in the cell cycle (cell cycle-related genes) (see the Methods section for additional details) were identified in each module. Additional file 5 lists cell cycle-related genes in the modules. Click here for file

Additional file 6 **Statistical results of correlation evaluation**. For each identified module pair, we evaluated gene correlations within and between modules. Additional file 6 lists the final results including module pairs with significant number of gene correlations and modules with significant number of gene correlations. Click here for file

Additional file 7 **Correlated genes of the identified module pairs**. Additional file 7 lists the correlated genes of each identified module pair. Click here for file

Additional file 8 **Statistical evaluation of the cooperation of the identified module pairs**. We evaluated the statistical significance of the cooperation of each module pair identified by our method and listed module pairs that significantly cooperate with genes functional in the cell cycle process or a specific phase. The column **Pair_ID **lists the unique identifier of each module pair. The column ***P*-value **lists the probability that the cooperation of a module pair associates with the cell cycle process or a specific phase. Click here for file

Additional file 9 **Phase-regulated gene set**. The phase-regulated gene set consisted of 416 genes with significant periodically changing expression identified by Cho *et al. *[45]. Click here for file

Additional file 10 **Cooperative relationship media ted by Cdc28 and phase-related regulators**. Additional file 10 lists cooperative module pairs that cooperate with essential regulators of the yeast cell cycle. The column **Regulator **lists regulators cooperating with a module pair. Click here for file

Additional file 11 **Modules containing Cdc28 and phase-related regulators**. We listed modules identified by our method that contain Cdc28 and phase-related regulators. Click here for file

Additional file 12 **Protein-protein interaction data**. Protein-protein interaction data for yeast were downloaded from the BioGRID database [13]. Click here for file
