# Supplementary material for: A novel method to identify cooperative functional modules: study of module coordination in the Saccharomyces cerevisiae cell cycle
Source: BMC Bioinformatics. 2011 Jul 12;12:281. doi: 10.1186/1471-2105-12-281 (PMC3143111; doi:10.1186/1471-2105-12-281)
Supplement: Additional file 1 — Supplementary discussions. Additional details of our method and discussions were described in Additional file 1. [file 1471-2105-12-281-S1.PDF]

Construction of A Weighted Physical Interaction Network (WPI Network)

In the first step (Figure 1A), to observe probable functional correlations among genes in the cell cycle, we constructed a co-expression gene network based on gene expression profiles during the yeast cell cycle from Cho *et al*. [1] (obtained from ExpressDB [2]). A node of the co-expression network represents a gene, and a link (or edges) represents the significant expression correlation between two genes. The Pearson correlation coefficients between the expression profiles of all $(N*(N-1))/2$ pairs of genes were estimated and ranked in descending order, where N is the number of genes in the microarray data. Pairs of genes with correlation scores above 0.683 or below -0.683 were selected and considered to be significant positive and negative co-expression, respectively. We set the threshold of correlation at 0.683 according to the t-test for non-zero correlation with a significance level of 0.005 and the degrees of freedom equal to 13 since our sample size is 15. For each gene, we generated a corresponding node, and for each significantly correlated gene pair, we generated a link between the corresponding nodes.

We then designed a weighted physical interaction (WPI) network using ChIP-chip data, protein-protein interaction data, and the co-expression network established in the previous step. The ChIP-chip data set was obtained from Harbison *et al*. [3] and contains regulatory interactions between 203 transcription factors and their target genes. Protein-protein interaction data identified with different experimental techniques for yeast consisting of 58,159 interactions between 5,450 proteins were downloaded from the BioGRID database [4]. Similar to the co-expression network, nodes in the WPI network represented genes, but links

represented protein-protein interactions from BioGRID and regulatory relationships from ChIP-chip data. Based on these data, we generated an undirected link for each protein-protein interaction and directed links from transcription factors to target genes. Finally, the degree (the number of links) of each gene in the co-expression network was assigned to each corresponding gene and represents the weight of each gene in the weighted physical interaction network (WPI network).

The WPI network was used to identify genes and the interactions among them that are likely to be significant and involved in the cell cycle and, importantly, to predict interactions associated with any two functional modules. The WPI network is shown as a weighted graph in which nodes represent genes and links represent protein-protein interactions or regulatory interactions between gene products. The weight of each node is a score that indicates the degree of necessity of that gene. The weight of each node was derived from the degree of the nodes in the aforementioned co-expression network. As significantly co-expressed genes tend to be functionally related, the Pearson correlation is a good scoring function used to evaluate the intensity of the functional correlation of a given pair of genes under specific conditions [5-8]. Furthermore, an essential role for most hub genes in a co-expression network has been shown to be more prevalent than with other genes in previous studies [9-12]; therefore, we used the number of co-expressed partners to estimate the probability that a gene is cell cycle-related. Links in the WPI network correspond to physical interactions in yeast and can be used to infer functional modules and cooperation among those modules. Therefore, we designed a depth-first search based algorithm to identify module pairs that show a high degree of cooperation in the WPI network.

Identification of Correlated Genes for Each Module Pair

We defined correlated genes of a cooperative module pair as genes that significantly cooperate with both modules. Cooperative interactions of module pairs can potentially be mediated by these correlated genes. These genes were used for analyzing the association between identified module pairs (Figure 1C).

To test whether gene *G* significantly cooperates with module *M*, we compared the number of interactions between *G* and the genes in *M* in the WPI network with that in each of 100 randomly generated networks using a method similar to a previous study [13] and shown in Figure 1B and Figure 2. If the number of links between *G* and genes contained in *M* in the weighted physical interaction network was higher than the 95th percentile of 100 random networks, gene *G* was regarded as being significantly associated with module M. Accordingly, for a correlated gene *x* of a module pair, the relationship between *x* and the two modules in the WPI network could be one of five types (see Figure 3):

(1) A significant number of undirected links between *x* and genes in one of the modules and a significant number of directed links from genes in the other module to *x*

(2) A significant number of undirected links between *x* and genes in one of the modules and a significant number of directed links from *x* to genes in the other modules

(3) A significant number of undirected links between *x* and genes in each of the two modules

(4) A significant number of directed links from genes in each of the two modules to *x*

(5) A significant number of directed links from *x* to genes in each of the two modules

These directed links represent transcriptional interactions and the undirected links represent protein-protein interactions between two genes. Therefore, these correlated genes of a cooperative module pair could be treated as important connectors that transfer signals between two modules and other functional components of yeast, in addition to connecting gene products of the two modules.

Evaluation of Correlations within and between Modules

To evaluate the correlations within and between modules of an identified module pair, we measured the significance of gene correlations within each module and between modules. We tested two types of correlation: physical interaction and co-expression. The significance of physical interaction within and between modules is measured by comparing the number of physical interactions within and between modules found in the weighted physical interaction network (WPI network) to that found in random networks. The procedure for evaluating the connectivity of physical interactions within genes of a module and between a pair of modules is described as follows:

1. Generate 100 random networks. Each random network is generated by randomly swapping interacted partners of the WPI network according to the method from a previous study [13].
2. Count physical interactions between genes of a module and crosstalk interactions between a module pair in each random network. Rank the number of physical interactions of a module and a module pair, respectively.
3. If the number physical interactions between genes contained in a module in the WPI network was higher than 95th percentile of 100 random networks, the correlation within the module was regarded as significant. If the number of crosstalk interactions between a module pair was higher than the 95th percentile

of 100 random networks, the correlation between a module pairs was regarded as significant.

To measure the significance of co-expressed correlations within and between modules of each identified module pair, we compared the number of co-expressed gene pairs within and between modules found in the cell cycle expression dataset from Cho *et al*. [1] with that found in randomized expression datasets. The procedure for evaluating co-expressed correlation within a module (or between modules) of an identified module pair is described as follows:

1. Repeatedly generated 1000 randomized expression datasets. A randomized expression dataset is generated by randomly shuffling the expression pattern of each gene.
2. Calculate the correlation score of each gene pairs within a module (or between modules). The correlation score of a gene pair is the Pearson correlation coefficients between the expression profiles of the gene pair. Only gene pairs with correlation scores above 0.683 or below -0.683 were considered to be co-expression gene pairs.
3. For each randomized expression dataset and the cell cycle expression dataset, we counted the number of co-expressed gene pairs within a module (or between modules) based on each randomized expression dataset and the cell cycle expression dataset, respectively.
4. If the number of co-expression gene pairs within a module (or between modules) was higher than the 95th percentile of 1000 randomized expression datasets, the proportion of gene correlations within a module (or between modules) was regarded as significant.

Structure and Properties of the Cooperative Module Network (CMN)

To analyze the functions and mechanisms of cooperative module pairs, the functions of each module were annotated using Gene Ontology (GO) [14] and identified cell cycle-related genes in each module using the cell cycle-related gene set. To predict function of a module, we annotated genes of the module to the *Saccharomyces* Genome Database Gene Ontology by the tool, GO Term Finder [14-16]. The tool calculates the significance of the enrichment of each GO term in a list of genes according to the hypergeometric distribution. We used the GO Term Finder to identify statistical significant enriched GO terms of a module (p-value < 0.01) as its annotation (Additional file 4). The GO term with the most significant p-value was chosen as the function of a module. The cell cycle-related gene set contains a total of 985 genes that are cell cycle-regulated or whose functions are annotated as cell cycle or DNA processing in MIPS [17, 18]. Genes contained in a module and in the cell cycle-related gene set were identified as cell cycle-related genes of the module (Additional file 5). Information about modules that link more than three modules in the cooperative module network is listed in Table 1. For each of these modules, Table 1 presents its function, a subset of cell cycle-related genes contained in it, and the number of genes in it. As shown, most modules, especially those in Table 1 (purple circles in Figure 4), were annotated as cell cycle-related or other specific functions with statistical significance (p-values < 0.01) (Table 1). Essential regulators that control the progress of the cell cycle, such as *CDC28*, cyclins, transcription factors, and checkpoint-related genes, were also identified in functionally corresponding modules. The main cooperative relationships among modules and the basic function of and implicit crosstalk interactions between modules

in the cell cycle are illustrated in Figure 4 and Table 1. For example, we found that 57 of 82 modules contain target genes of Cdc28 [19, 20]. These results provide evidence for potential cooperative interactions between modules containing *CDC28* and other modules. Table 1 shows that the degree number of module 0 (response to DNA damage stimulus) was the highest and ranged up to 34. This module coordinates with most components and is a central hub in the cooperative module network. The importance of this module is explicitly demonstrated by the genes contained in it and its interactions with other modules (Figure 4 and Table 1). Specifically, this module contains genes whose products sense DNA damage, activate the DNA repair system and pass this signal to other functional components such as modules involved in DNA replication [e.g., module 10 (maintenance of fidelity during DNA-dependent DNA replication), and module 12 (DNA replication initiation)] to induce appropriate cell responses. Module 2 (Golgi vesicle transport) and module 30 (cell morphogenesis) contain the second and the third largest numbers of functional components. These two modules are also hubs connected to various functional modules. Taking module 30 (cell morphogenesis) as another example, some essential cyclin genes such as *CLN4* and *CDC28* are included in this module. These results are consistent with previous studies demonstrating that yeast cyclin-dependent kinases (CDKs) are involved in driving cell morphogenesis [21, 22]. Another important feature of the cooperative module network is that modules working in the same cell cycle phase tend to form a highly connected sub-network. For example, M phase-related modules such as modules 36 (spindle organization), 37 (nuclear division) and 17 (chromosome segregation) show strong connectivity in the cooperative module network and are highly associated with each other (Figure 4). According to the interactions in the cooperative module network and the genes shown in Table 1, we identified crucial genes that mediate cooperative interactions in the cell cycle, such as genes involved in

DNA damage repair and mitotic checkpoints.

Communication Mechanisms and Functions of Phase-related Cooperative Modules

To discover the most essential cooperating module pairs and to determine their functions, we ranked the module pairs according to the number of their correlated genes that are regulated in a specific cell cycle phase. Genes regulated in a specific phase were identified with the phase-regulated gene set from Cho *et al.* [1]. In this way, the top three module pairs were chosen for each phase of the cell cycle (Table 2). References of demonstrated cooperative associations among genes were listed in Table A1.

As shown in Table 2, cell cycle phase transitions are accompanied by changes in the main functional modules and their interactions. Cooperative relationships of modules in each phase of the cell cycle seem to be established differently by different gene interactions within modules. For example, genes that function in response to DNA damage stimulus (genes in module 0) can communicate with genes related to mismatch repair (genes in module 10) via Pol32 (late G1 phase in Table 2) or with mitosis-related genes (genes in module 3) at the S and G2/M checkpoints via *Rad53* (S and G2 phase in Table 2). Moreover, we found that signal transduction among modules occurs mainly through Cdc28 and that Cdc28 associates with different cyclins, transcription factors and genes regulated in different phases to promote cell cycle progression (early G1, late G1, G2, and M phase in Table 2). In addition, Rad9 and Rad53 play important roles in communication between Module 0 (response to DNA damage stimulus) and other checkpoint-dependent modules (early G1, late G1, S, and G2 phase in Table 2).

In Table 2, module 30 (cell morphogenesis) and module 32 (regulation of cell division) are shown to be key cooperating partners in early G1 phase. Crosstalk and cooperative interactions between these two modules and others is primarily mediated by Cdc28 (early G1 and M phase in Table 2). In addition, the DNA damage checkpoint mechanism is established by associations with genes in module 0 (response to DNA damage stimulus) (early G1 phase in Table 2). The cooperative relationships of modules in late G1 rely on interactions in module 0 (response to DNA damage stimulus) (late G1 phase in Table 2). Genes related to DNA damage checkpoints, repair systems and other DNA replication functions are expressed in late G1 phase. Module 0 (response to DNA damage stimulus) contains genes for sensing DNA damage that recruit various repair systems to repair the damage. The presence of this module was observed in early G1, late G1, S and G2 phases (Table 2). Module 0 (response to DNA damage stimulus) cooperates with diverse functional modules and is matched with each checkpoint process (No.7-18 and No.23 in Table A1). For example, the G2/M checkpoint is accomplished by module 0 (response to DNA damage stimulus) and module 3 (mitosis) (S phase in Table 2). Module 0 (response to DNA damage stimulus) interacts with chromosome segregation-related genes from module 3 (mitosis) for the G2/M checkpoint and DNA repair by associating with the S phase-regulated gene *PDS1* (S phase in Table 2). In the S phase, associations among the components for chromosome segregation, such as the kinetochore complex and spindle pole body, are traced by interactions between modules 37 and 17 (S phase in Table 2). Through G2 and M phases, the important connector Cdc28 is still active and coordinates with cyclins and regulators to promote cell cycle processes (G2 and M phase in Table 2).

Module Crosstalk Networks Under the Regulation of Cdc28,

Phase-related Cyclins, Cell Division Cycle Genes (CDC Genes) and Transcription Factors

To present a map of cooperative regulation and interactive mechanisms between identified modules in the cell cycle, we constructed relationship graphs for each phase by combining correlated genes of module pairs and phase-related regulators (see Table 3). In these relationship graphs, each identified module and regulators not located in any of these modules are represented by a node. To present significant associations, we only consider regulators that are correlated genes of a module pair and relationships among correlated genes and their correlated module pairs (Figure 3). Direct connections mediated by regulators between either two different modules or a module and a regulator that are not located in any module are represented by a link. Because we only consider relations between identified module pairs and their correlated genes (Figure 3), a link indicates a significant interacting association. The links of each relationship graph can be directed or undirected depending on the type of relation (Figure 3). Directed links represent transcriptional associations and undirected links represent protein-protein associations. Consider a cooperative module pair, module *m1* and module *m2*, and the regulator *x* (one of the regulators listed in Table 3). If *x* is a correlated gene of the module pair that regulates (by either transcriptional or protein-protein interactions) or is regulated by a significant number of genes in module *m2*, a link will be generated between either the module containing *x* or *x* and module *m2* according to the type of interaction. Similarly, a link will be generated between either the module containing *x* or *x* and module *m1*. For example, if *x* is contained in *m1* and is transcriptionally regulated by a significant number of genes in *m2*, a directed link will be generated from *m2* to *m1*. Repeated links were summarized to one link. For example, if a module contains at least one correlated

genes that associate with the other module by a significant number of protein-protein interactions, we only generate a link to represent the relationship between modules. Finally, we excluded modules without a link from each relationship graph. Therefore, in each relationship graph, a link between two modules can be treated as a significant crosstalk relationship. Using this approach, we were able to explain or directly discover the regulation of the identified cooperative module pairs and the cell cycle regulators in each phase.

Figures 6A, 7A, 8A, and 9A show interacting relationships between modules controlled by specific transcription factors in G1, S, G2 and M phases. Figures 6B, 7B, 8B, and 9B show the CDC genes, cyclins and Cdc28-associated crosstalk relationships of G1, S, G2 and M phases. In these graphs, module pairs mediated by these regulators and modules that contain these regulators were investigated (detailed information is in Additional file 11 and 12). We also summarized the crosstalk relationships according to related stages in the cell cycle, and the most essential and specific associations in each phase. The most essential and specific associations of each crosstalk relationship graph that were previously reported are shown in Figure 10. The associations mediated by Cdc28, cyclins, and CDC genes during G2 and M phases were merged into the graph of G2 phase in Figure 10. A total of 59 module pairs that correlate with these regulators were identified (see Additional file 11). Moreover, significant cross-links mediated by these regulators were discovered among 25 module pairs. Figure 11 shows the number of identified module pairs mediated by each regulator. In Figures 6, 7, 8, and 9, a node is mapped to a unique module or a regulator (transcription factors, CDC genes, cyclins or Cdc28) not contained in any of identified modules, and a link represents a significant association that occurs either directly via regulatory interactions or via protein-protein interactions through a regulator. To easily distinguish modules and genes in the

crosstalk relationship graphs, we used yellow circles to represent modules and green circles to represent regulators. The size of the yellow circles is proportional to the number of genes within the modules. Consider a cooperative module pair (modules *X* and *Y*) and a regulator *Z*. When *Z* is a correlated gene of the module pair, two links (directed or undirected) will be generated to link either the module containing the regulator *Z* or the regulator *Z* to each of the two modules. Thus, by identifying phase-specific module interactions involved in cell cycle regulation, we can further determine the influences and functions of module interactions and regulators in controlling the cell cycle (see Figure 10).

When only connections via protein-protein interactions were considered, both the relationship graphs of Cdc28 and CDC genes and the relationship graph of G1 phase-related transcription factors showed a compact connectivity of modules (Figure 6 and 10). This type of connectivity implies that modules display direct crosstalk with each other. For example, modules 0 (response to DNA damage stimulus), 32 (regulation of cell division), and 42 (G1/S transition of mitotic cell cycle and interphase) all contain Cdc28, and these modules connect to each other to form a clique-like subgraph in the relationship graph of Cdc28, cyclins, and CDC genes (Figure 6B and G1 phase of Figure 10). As shown in the relationship graph of Cdc28, cyclins, and CDC genes, a group of interconnecting modules consisting of modules that connect to Mbp1 was also discovered in the related graph of G1-phase transcription factors (Figure 6A and G1 phase in Figure 10). These associations are consistent with previous studies [23, 24]. When we considered only regulatory connections, however, modules tended to be co-regulated by only one gene or module. Mbp1 is an example of this kind of connectivity (Figure 6A and Figure 10). Interestingly, the G1 relationship graph of Cdc28 and the CDC genes is rather different from the graph of phase-related transcription factors and displays only Cln1

with regulatory connections from modules 0 (response to DNA damage stimulus), 30 (cell morphogenesis) and 32 (regulation of cell division) (Figure 6B). These relationships suggest that cell cycle progression could be control by these modules via Cln1 (G1 phase of Figure 10 and No.2 in Table A2). In the relationship graph, modules 1 (regulation of cell cycle), 14 (regulation of transcription during G2/M phase), 30 (cell morphogenesis), 32 (regulation of cell division), 42 (G1/S transition of mitotic cell cycle and interphase), and 79 (spindle checkpoint) comprise a highly connected subgraph due to protein-protein interactions with Cdc28, and they provide evidence for the regulation of Cdc28 (No.3 in Table A2). Moreover, modules 1 (regulation of cell cycle), 30 (cell morphogenesis), 32 (regulation of cell division), and 42 (G1/S transition of mitotic cell cycle and interphase) interact with Cln1, and we can infer that these modules have relationships with Cln1, Cln2, Cln3, and Cdc28 (6B, G1 phase in Figure 10, and No.2 in Table A2).

Given that most transcription factors in the relationship graph of G1 regulate genes during G1/S, the relationship graph of S is similar to that of G1 (Figure 6A and 7A). Obvious differences between the relationship graphs of G1 and S phase include the associations through Ndd1, which is contained in module 14 (regulation of transcription during G2/M phase) (Figure 7A and S phase in Figure 10). In addition to its essential roles in G2/M, Ndd1 is also regulated during S phase and is critical for the transcription of S phase-specific genes [25]. Regulatory relationships between module 14 and other functional components are shown in the relationship graph and include possible interactions between Ndd1 and transcription factors in other modules such as Swi4 in module 30 (cell morphogenesis) (Figure 7A and S phase in Figure 10, and No.5 in Table A2). According to our results, most correlations between modules occur through Cdc7 and Cdc24. Cdc7 is contained in module 12 (DNA-dependent DNA replication) and functions in DNA replication (Figure 7B and S phase in Figure

10) [26, 27]. It mediates signals between DNA replication-related modules such as modules 0 (response to DNA damage stimulus) and 10 (maintenance of fidelity during DNA-dependent DNA replication) (Figure 7B, S phase in Figure 10, and No.6 in Table A2). Cdc24 is involved in cytoskeletal rearrangements and is essential for cell morphogenesis [28, 29]. Crosstalk between module 77 (regulation of nuclear division) and other functional modules such as module 30 (cell morphogenesis), is dependent on Cdc24 (Figure 7B and S phase in Figure 10, and No.7 in Table A2).

In the G2 phase relationship graph of CDC genes and Cdc28, most of the relationships are primarily due to interactions involving Cdc28, G2/M related-cyclins and Cdc28 substrates (Figure 8B, 9B, and G2 phase in Figure 10). Some of these module associations involving interactions of Cdc28, Clb2, Clb3, and other G2/M related transcription factors in our relationship graphs have been demonstrated previously (G2 phase in Figure 10 and No.8 in Table A2). Furthermore, the dense connections of the graphs (G2 phase in Figure 10, Figure 8A and 9A) also indicate significant interactions between these modules. Regulatory relationships of reported functional modules in G2 phase and M phase are mediated by module 14 (regulation of transcription during G2/M phase) and Mcm1. In these graphs, we observed that the main transcription factors involved in the regulation of G2/M-related genes (Fkh1, Fkh2 and Ndd1) are contained in module 14 (regulation of transcription during G2/M phase) and regulate genes in diverse modules (G2 phase in Figure 10 and No.9 in Table A2). We also inferred from the graphs that these regulators cooperate with Mcm1 are highly associated with transcription factors of module 14 (regulation of transcription during G2/M phase), Cdc28, and the CDC genes in G2/M phase (8A, 9A, G2 phase of Figure 10, and No.10 in Table A2). Direct associations of these regulators with target genes are reflected in links between Mcm1 and module 14. Important regulatory roles of Mcm1 and module 14 are shown by direct links to G2 and M

phase-related modules that function in cell morphology such as module 30 for cell morphology and module 57 for chromosome segregation (Figure 8A, 9A, G2 and M phase of Figure 10, and No.11 in Table A2).

Table A1. References for cooperative interactions.

No.	Genes	Ref.
1	*RAD23, RAD51*	[30]
2	*CLB2, FKH1, genes encoding proteasome*	[31]
3	*SWI4, CDC28, CLN2, CLN3*	[32]
4	*BNI1, FUS1, FUS3, STE5, FAR1, CLNs, CDC28*	[33]
5	*SWE1, CDC28, CLN2, CDC4, CDC34, CDC6*	[34]
6	*CDC34, CLN2, BNI1, SLT2, PKC1, FAR1, CDC6*	[35]
7	*CLN2, CLN3, RAD9, RAD53, MRC1, SWI6, CDC45, MEC1, SWI4*	[36]
8	*STB1, SWI6*	[24]
9	*RAD6, RAD18, CDC28, HO, SWI4*	[37]
10	*CDC28, SWE1, CLB5, CLB6, HO*	[38]
11	*RAD53, CLB5, CLB6, CDC28*	[39]
12	*RAD1, RAD14, RAD6, CDC9*	[40]
13	*RAD53, RAD14, RAD52*	[41]
14	*POL30, PLO32, MSH2, MSH3, MSH6, MLH1*	[42]
15	*RAD9, EXO1*	[43]
16	*PLO30, PLO32, MSH2, MLH1*	[44]
17	*CIN8, KIP3, MAD2, RAD53, DUN1, MEC1, PDS1*	[45, 46]
18	*PDS1, CDH1, RAD53*	[47]
19	*SPC24, DAM1, CBF3, MTW1, NDC80, SPC24, SPC110, CBF2*	[48]
20	*SPC24, DAM1, MTW1, NNF1, NSL1, DSN1, SPC24, SPC110*	[49]
21	*BIM1, KAR9, BIK1*	[50]
22	*BIM1, CIN8, BIK1*	[51]
23	*MOB1, SIC1, CDC28, DBF2, LTE1, CLB2, DBF20*	[52]
24	*GCN5, HTZ1*	[53]
25	*GCN5, SPT8, RAD53, ASF1, MEC1*	[54]
26	*RAD51, RAD53, HTZ1*	[55]

27	*CDC28, CDC34,CDC4, SWE1, PKC1, CHS2*	[20]
28	*ELM1, SWE1, CDC28, CDC4, CDC34*	[56]
29	*CDC28, SWI5, ACE2, FKH2, FKH1, NDD1, CDC5, CLB3*	[57]
30	*SWI5, PLC2, PHO85, CDC28*	[58]
31	*CLB3, CLB5, SIC1, CDC28, CLB1, CLB4, CDC20*	[59]
32	*BUB3, CDC20, CDC28*	[60]
33	*CDC28, CLN1, CLN2, FUS3, MSG5, BUD2*	[20]
34	*SLT2, FUS3, MSG5*	[61]
35	*CDC12, CLN1, CLN2, BUD2*	[62]

Table A1 listed references of demonstrated cooperative associations among genes. Associated genes confirmed by each reference were listed in the second column.

Table A2. References for crosstalk associations.

Phase	No.	Regulator	Ref
G1	1	*STB1, MBP1, SWI6, SWI4, CLN1, CLN2, CLN3, CDC28*	[23, 24]
	2	*CLN1, CLN2, CLN3, CDC28*	[63-67]
	3	*CDC28*	[19, 38, 63, 68, 69]
S	4	*MBP1, SWI6, SWI4, CLN1, CLN2, CLB6, CLB5*	[66]
	5	*NDD1*	[70]
	6	*CDC7*	[26, 27]
	7	*CDC24*	[28, 29]
G2	8	*NDD1, FKH1, FKH2, CLB1, CLB2, CLB3, CDC28, CDC5*	[57, 71]
	9	*MCM1, NDD1, FKH1, FKH2, ACE2*	[72-75]
	10	*MCM1, NDD1, FKH1, FKH2, ACE2, SWI5, CDC28, CDC5*	[57, 71, 76, 77]
M	11	*MCM1, NDD1, FKH1, FKH2, ACE2*	[72]

Table A2 listed references of demonstrated crosstalk associations (Figure 10). Associated regulators confirmed by each reference were listed in the third column.

References:

1. Cho RJ, Campbell MJ, Winzeler EA, Steinmetz L, Conway A, Wodicka L,

Wolfsberg TG, Gabrielian AE, Landsman D, Lockhart DJ *et al*: **A genome-wide transcriptional analysis of the mitotic cell cycle**. *Molecular cell* 1998, **2**:65-73.

2. Aach J, Rindone W, Church GM: **Systematic management and analysis of yeast gene expression data**. *Genome Research* 2000, **10**(4):431-445.
3. Harbison CT, Gordon DB, Lee TI, Rinaldi NJ, Macisaac KD, Danford TW, Hannett NM, Tagne JB, Reynolds DB, Yoo J *et al*: **Transcriptional regulatory code of a eukaryotic genome.** *Nature* 2004, **431**:99-104.
4. Stark C, Breitkreutz BJ, Reguly T BL, Breitkreutz A, Tyers M: **BioGRID: a general repository for interaction datasets.** *Nucleic Acids Research* 2006, **34**:D535-D539.
5. Eisen MB, Spellman PT, Brown PO, Botstein D: **Cluster analysis and display of genome-wide expression patterns**. *Proc Natl Acad Sci U S A* 1998, **95**(25):14863-14868.
6. Schmitt WA Jr, Raab RM, Stephanopoulos G: **Elucidation of gene interaction networks through time-lagged correlation analysis of transcriptional data.** *Genome Research* 2004, **14**(8):1654-1663.
7. Chen Y, Xu D: **Global protein function annotation through mining genome-scale data in yeast *Saccharomyces cerevisiae*.** *Nucleic Acids Res* 2004, **32**:6414-6424.
8. Choi JK, Yu U, Yoo OJ, Kim S: **Differential coexpression analysis using microarray data and its application to human cancer.** *Bioinformatics* 2005, **21**(24):4348-4355.
9. Horvath S, Dong J: **Geometric Interpretation of Gene Coexpression Network Analysis.** *PLoS Comput Biol* 2008, **4**(8):e1000117.
10. Saris CG, Horvath S, van Vught PW, van Es MA, Blauw HM, Fuller TF, Langfelder P, Deyoung J, Wokke JH, Veldink JH *et al*: **Weighted gene co-expression network analysis of the peripheral blood from Amyotrophic Lateral Sclerosis patients.** *BMC Genomics* 2009, **10**(1):405.
11. Mao L, Van Hemert JL, Dash S, Dickerson JA: **Arabidopsis gene co-expression network and its functional modules.** *BMC Bioinformatics* 2009, **10**:346.
12. Carter SL, Brechbühler CM, Griffin M, Bond AT: **Gene co-expression network topology provides a framework for molecular characterization of cellular state.** *Bioinformatics* 2004, **20**(14):2242-2250.
13. Milo R, Shen-Orr S, Itzkovitz S, Kashtan N, Chklovskii D, Alon U: **Network motifs: simple building blocks of complex networks**. *Science* 2002, **298**(5594):824-827.

14. Ashburner M, Ball CA, Blake JA, Botstein D, Butler H, Cherry JM, Davis AP, Dolinski K, Dwight SS, Eppig JT et al: **Gene ontology: tool for the unification of biology. The Gene Ontology Consortium.** *Nature genetics* 2000, **25**:25-29.
15. Christie KR, Weng S, Balakrishnan R, Costanzo MC, Dolinski K, Dwight SS, Engel SR, Feierbach B, Fisk DG, Hirschman JE *et al*: ***Saccharomyces* Genome Database (SGD) provides tools to identify and analyze sequences from *Saccharomyces cerevisiae* and related sequences from other organisms**. *Nucleic Acids Res* 2004, **32**(Database issue):D311-D314.
16. Boyle EI, Weng S, Gollub J, Jin H, Botstein D, Cherry JM, Sherlock G: **GO::TermFinder--open source software for accessing Gene Ontology information and finding significantly enriched Gene Ontology terms associated with a list of genes.** *Bioinformatics* 2004, **20**(18):3710-3715.
17. Mewes HW, Frishman D, Güldener U, Mannhaupt G, Mayer K, Mokrejs M, Morgenstern B, Münsterkötter M, Rudd S, Weil B: **MIPS: A database for genomes and protein sequences**. *Nucleic Acids Research* 2002, **30**:31-34.
18. de Lichtenberg U, Jensen LJ, Fausbøl A, Jensen TS, Bork P, Brunak S: **Comparison of computational methods for the identification of cell cycle-regulated genes.** *Bioinformatics* 2005, **21**:1164-1171.
19. Ubersax JA, Woodbury EL, Quang PN, Paraz M, Blethrow JD, Shah K, Shokat KM, Morgan DO: **Targets of the cyclin-dependent kinase Cdk1.** *Nature* 2003, **425**(6960):859-864.
20. Enserink JM, Kolodner RD: **An overview of Cdk1-controlled targets and processes**. *Cell Div* 2010, **5**:11.
21. Lew DJ, Reed SI: **Morphogenesis in the yeast cell cycle: regulation by Cdc28 and cyclins.** *J Cell Biol* 1993, **120**(6):1305-1320.
22. Moffat J, Andrews B: **Late-G1 cyclin–CDK activity is essential for control of cell morphogenesis in budding yeast.** *Nature Cell Biology* 2003, **6**(59-66).
23. Horak CE, Luscombe NM, Qian J, Bertone P, Piccirrillo S, Gerstein M, Snyder M: **Complex transcriptional circuitry at the G1/S transition in *Saccharomyces cerevisiae***. *Genes Dev* 2002, **16**(23):3017-3013.
24. de Bruin RA, Kalashnikova TI, Wittenberg C: **Stb1 collaborates with other regulators to modulate the G1-specific transcriptional circuit**. *Mol Biol Cell* 2008, **28**(22):6919-6928.
25. Loy CJ, Lydall D, Surana U: **NDD1, a high-dosage suppressor of cdc28-1N, is essential for expression of a subset of late-S-phase-specific genes in *Saccharomyces cerevisiae*.** *Mol Biol Cell* 1999, **19**(5):3312-3327.
26. Bousset K, Diffley JF: **The Cdc7 protein kinase is required for origin firing**

during S phase. *Genes Dev* 1998, **12**(4):480-490.

27. Pessoa-Brandão L, Sclafani RA: **CDC7/DBF4 Functions in the Translesion Synthesis Branch of the RAD6 Epistasis Group in *Saccharomyces cerevisiae***. *Genetics* 2004, **167**(4):1597-1610.

28. Chenevert J, Valtz N, Herskowitz I: **Identification of genes required for normal pheromone-induced cell polarization in *Saccharomyces cerevisiae*.** *Genetics* 1994, **136**(4):1287-1296.

29. Sloat BF, Pringle JR: **A mutant of yeast defective in cellular morphogenesis.** *Science* 1978, **200**(4346):1171-1173.

30. Wade SL, Poorey K, Bekiranov S, Auble DT: **The Snf1 kinase and proteasome-associated Rad23 regulate UV-responsive gene expression**. *EMBO J* 2009, **28**(19):2919-2931.

31. Jorgensen P, Tyers M: **The fork'ed path to mitosis.** *Genome Biol* 2000, **1**(3):REVIEWS1022.

32. Koch C, Schleiffer A, Ammerer G, Nasmyth K: **Switching transcription on and off during the yeast cell cycle: Cln/Cdc28 kinases activate bound transcription factor SBF (Swi4/Swi6) at start, whereas Clb/Cdc28 kinases displace it from the promoter in G2**. *Genes Dev* 1996, **10**(2):129-141.

33. Yu L, Qi M, Sheff MA, Elion EA: **Counteractive control of polarized morphogenesis during mating by mitogen-activated protein kinase Fus3 and G1 cyclin-dependent kinase.** *Mol Biol Cell* 2008, **19**(4):1739-1752.

34. Kaiser P, Sia RA, Bardes EG, Lew DJ, Reed SI: **Cdc34 and the F-box protein Met30 are required for degradation of the Cdk-inhibitory kinase Swe1.** *Genes Dev* 1998, **12**(16):2587-2597.

35. Varelas X, Stuart D, Ellison MJ, Ptak C.: **The Cdc34/SCF ubiquitination complex mediates *Saccharomyces cerevisiae* cell wall integrity.** *Genetics* 2006, **174**(4):1825-1839.

36. Sidorova JM, Breeden LL: **Rad53-dependent phosphorylation of Swi6 and down-regulation of CLN1 and CLN2 transcription occur in response to DNA damage in *Saccharomyces cerevisiae*.** *Genes Dev* 1997, **11**(22):3032-3045.

37. Kaplun L, Ivantsiv Y, Kornitzer D, Raveh D: **Functions of the DNA damage response pathway target Ho endonuclease of yeast for degradation via the ubiquitin-26S proteasome system**. *Proc Natl Acad Sci U S A* 2000, **97**(18):10077-10082.

38. Hu F, Aparicio OM: **Swe1 regulation and transcriptional control restrict the activity of mitotic cyclins toward replication proteins in *Saccharomyces cerevisiae***. *Proc Natl Acad Sci U S A* 2005,

102(25):8910-8915.

39. Gibson DG, Aparicio JG, Hu F, Aparicio OM: **Diminished S-phase cyclin-dependent kinase function elicits vital Rad53-dependent checkpoint responses in *Saccharomyces cerevisiae***. *Mol Biol Cell* 2004, **24**(23):10208-10222.

40. Guzder SN, Sommers CH, Prakash L, Prakash S: **Complex formation with damage recognition protein Rad14 is essential for *Saccharomyces cerevisiae* Rad1-Rad10 nuclease to perform its function in nucleotide excision repair in vivo.** *Mol Biol Cell* 2006, **26**(3):1135-1141.

41. Neecke H, Lucchini G, Longhese MP: **Cell cycle progression in the presence of irreparable DNA damage is controlled by a Mec1- and Rad53-dependent checkpoint in budding yeast.** *EMBO J* 1999, **18**(16):4485-4497.

42. Amin NS, Nguyen MN, Oh S, Kolodner RD: **exo1-Dependent mutator mutations: model system for studying functional interactions in mismatch repair**. *Mol Cell Biol* 2001, **21**(15):5142-5155.

43. Morin I, Ngo HP, Greenall A, Zubko MK, Morrice N, Lydall D: **Checkpoint-dependent phosphorylation of Exo1 modulates the DNA damage response**. *EMBO J* 2008, **27**(18):2400-2410.

44. Umar A, Buermeyer AB, Simon JA, Thomas DC, Clark AB, Liskay RM, Kunkel TA: **Requirement for PCNA in DNA mismatch repair at a step preceding DNA resynthesis**. *Cell* 1996, **87**(1):65-73.

45. Krishnan V, Nirantar S, Crasta K, Cheng AY, Surana U: **DNA replication checkpoint prevents precocious chromosome segregation by regulating spindle behavior**. *Mol Cell* 2004, **16**(5):687-700.

46. Gardner R, Putnam CW, Weinert T: **RAD53, DUN1 and PDS1 define two parallel G2/M checkpoint pathways in budding yeast**. *EMBO J* 1999, **18**(11):3173-3185.

47. Cohen-Fix O, Koshland D: **The anaphase inhibitor of *Saccharomyces cerevisiae* Pds1p is a target of the DNA damage checkpoint pathway**. *Proc Natl Acad Sci U S A* 1997, **94**(26):14361-14366.

48. Janke C, Ortiz J, Lechner J, Shevchenko A, Shevchenko A, Magiera MM, Schramm C, Schiebel E: **The budding yeast proteins Spc24p and Spc25p interact with Ndc80p and Nuf2p at the kinetochore and are important for kinetochore clustering and checkpoint control.** *EMBO J* 2001, **20**(4):777-791.

49. Euskirchen GM: **Nnf1p, Dsn1p, Mtw1p, and Nsl1p: a new group of proteins important for chromosome segregation in *Saccharomyces***

cerevisiae. *Eukaryot Cell* 2002, **1**(2):229-240.
50. Miller RK, Cheng SC, Rose MD: **Bim1p/Yeb1p mediates the Kar9p-dependent cortical attachment of cytoplasmic microtubules**. *Mol Biol Cell* 2000, **11**(9):2949-2959.
51. Shimogawa MM, Wargacki MM, Muller EG, Davis TN: **Laterally attached kinetochores recruit the checkpoint protein Bub1, but satisfy the spindle checkpoint.** *cell cycle* 2010, **9**(17):3619-3628.
52. Mah AS, Jang J, Deshaies RJ: **Protein kinase Cdc15 activates the Dbf2-Mob1 kinase complex**. *Proc Natl Acad Sci U S A* 2001, **98**(13):7325-7330.
53. Eirín-López J, Ausió J: **H2A.Z-Mediated Genome-Wide Chromatin Specialization.** *Curr Genomics* 2007, **8**(1):59-66.
54. Burgess RJ, Zhou H, Han J, Zhang Z: **A role for Gcn5 in replication-coupled nucleosome assembly.** *Mol Cell* 2010, **37**(4):469-480.
55. Kalocsay M, Hiller NJ, Jentsch S: **Chromosome-wide Rad51 spreading and SUMO-H2A.Z-dependent chromosome fixation in response to a persistent DNA double-strand break.** *Mol Cell* 2009, **33**(3):335-343.
56. Bouquin N, Barral Y, Courbeyrette R, Blondel M, Snyder M, Mann C: **Regulation of cytokinesis by the Elm1 protein kinase in *Saccharomyces cerevisiae***. *J Cell Sci* 2000, **113**(Pt 8):1435-1445.
57. Reynolds D, Shi BJ, McLean C, Katsis F, Kemp B, Dalton S: **Recruitment of Thr 319-phosphorylated Ndd1p to the FHA domain of Fkh2p requires Clb kinase activity: a mechanism for CLB cluster gene activation.** *Genes Dev* 2003, **17**(14):1789-1802.
58. Aerne BL, Johnson AL, Toyn JH, Johnston LH: **Swi5 controls a novel wave of cyclin synthesis in late mitosis**. *Mol Biol Cell* 1998, **9**(4):945-956.
59. Rudner AD, Murray AW: **Phosphorylation by Cdc28 activates the Cdc20-dependent activity of the anaphase-promoting complex.** *J Cell Biol* 2000, **149**(7):1377-1390.
60. Larsen NA, Al-Bassam J, Wei RR, Harrison SC: **Structural analysis of Bub3 interactions in the mitotic spindle checkpoint.** *Proc Natl Acad Sci U S A* 2007, **104**(4):1021-1026.
61. Marín MJ, Flández M, Bermejo C, Arroyo J, Martín H, Molina M: **Different modulation of the outputs of yeast MAPK-mediated pathways by distinct stimuli and isoforms of the dual-specificity phosphatase Msg5.** *Mol Genet Genomics* 2009, **281**(3):345-359.
62. Park HO, Sanson A, Herskowitz I: **Localization of Bud2p, a GTPase-activating protein necessary for programming cell polarity in**

yeast to the presumptive bud site. *Genes Dev* 1999, **13**(15):1912-1917.

63. Wittenberg C, Sugimoto K, Reed SI: **G1-specific cyclins of S. cerevisiae: cell cycle periodicity, regulation by mating pheromone, and association with the p34CDC28 protein kinase.** *Cell* 1990, **62**(2):225-237.

64. Moffat J, Andrews B: **Late-G1 cyclin-CDK activity is essential for control of cell morphogenesis in budding yeast.** *Nat Cell Biol* 2004, **6**(1):59-66.

65. Saracino F, Bassler J, Muzzini D, Hurt E, Agostoni Carbone ML: **The yeast kinase Swe1 is required for proper entry into cell cycle after arrest due to ribosome biogenesis and protein synthesis defects**. *Cell Cycle* 2004, **3**(5):648-654.

66. Bean JM, Siggia ED, Cross FR: **High functional overlap between MluI cell-cycle box binding factor and Swi4/6 cell-cycle box binding factor in the G1/S transcriptional program in *Saccharomyces cerevisiae*.** *Genetics* 2005, **171**(1):49-61.

67. Dirick L, Böhm T, Nasmyth K: **Roles and regulation of Cln-Cdc28 kinases at the start of the cell cycle of *Saccharomyces cerevisiae***. *EMBO J* 1995, **14**(19):4803-4813.

68. Manderson EN, Malleshaiah M, Michnick SW: **A novel genetic screen implicates Elm1 in the inactivation of the yeast transcription factor SBF**. *PLos One* 2008, **3**(1):e1500.

69. Donovan JD, Toyn JH, Johnson AL, Johnston LH: **P40SDB25, a putative CDK inhibitor, has a role in the M/G1 transition in *Saccharomyces cerevisiae*.** *Genes Dev* 1994, **8**(14).

70. Simon I, Barnett J, Hannett N, Harbison CT, Rinaldi NJ, Volkert TL, Wyrick JJ, Zeitlinger J, Gifford DK, Jaakkola TS *et al*: **Serial regulation of transcriptional regulators in the yeast cell cycle**. *Cell* 2001, **106**(6):697-708.

71. Veis J, Klug H, Koranda M, Ammerer G: **Activation of the G2/M-specific gene CLB2 requires multiple cell cycle signals**. *Mol Biol Cell* 2007, **27**(23):8364-8373.

72. Hollenhorst PC, Bose ME, Mielke MR, Müller U, Fox CA: **Forkhead genes in transcriptional silencing, cell morphology and the cell cycle. Overlapping and distinct functions for FKH1 and FKH2 in *Saccharomyces cerevisiae***. *Genetics* 2000, **154**(4):1533-1548.

73. Zhu G, Spellman PT, Volpe T, Brown PO, Botstein D, Davis TN, Futcher B: **Two yeast forkhead genes regulate the cell cycle and pseudohyphal growth**. *Nature* 2000, **406**(6791):90-94.

74. O'Conallain C, Doolin MT, Taggart C, Thornton F, Butler G: **Regulated nuclear localisation of the yeast transcription factor Ace2p controls**

expression of chitinase (CTS1) in *Saccharomyces cerevisiae*. *Mol Gen Genet* 1999, **262**(2):275-282.

75. Darieva Z, Pic-Taylor A, Boros J, Spanos A, Geymonat M, Reece RJ, Sedgwick SG, Sharrocks AD, Morgan BA: **Cell cycle-regulated transcription through the FHA domain of Fkh2p and the coactivator Ndd1p**. *Gurr Biol* 2003, **13**(19):1740-1745.

76. Althoefer H, Schleiffer A, Wassmann K, Nordheim A, Ammerer G: **Mcm1 is required to coordinate G2-specific transcription in *Saccharomyces cerevisiae***. *Mol Biol Cell* 1995, **15**(11):5917-5928.

77. Kumar R, Reynolds DM, Shevchenko A, Shevchenko A, Goldstone SD, Dalton S: **Forkhead transcription factors, Fkh1p and Fkh2p, collaborate with Mcm1p to control transcription required for M-phase**. *Curr Biol* 2000, **10**(15):896-906.
