# Supplementary material for: A novel method to identify cooperative functional modules: study of module coordination in the Saccharomyces cerevisiae cell cycle
Source: BMC Bioinformatics. 2011 Jul 12;12:281. doi: 10.1186/1471-2105-12-281 (PMC3143111; doi:10.1186/1471-2105-12-281)
Supplement: Additional file 3 — Functional annotation results o f the identified modules. Additional file 3 lists functional annotation results of the 82 modules. We annotated functions of the identified modules from biological processes of Gene Ontology and listed the most significant function of each module. [file 1471-2105-12-281-S3.PDF]

Table S1. Functional annotation results of the identified modules.

Module ID	Function	*P*-value
0	response to DNA damage stimulus	3.93e-33
1	regulation of cell cycle	0.000154
2	vesicle-mediated transport	5.21e-12
3	mitosis	4.92e-14
4	ubiquitin-dependent protein catabolism	2.20e-30
5	protein insertion into mitochondrial membrane	4.69e-05
6	mitochondrial translation	8.54e-12
7	DNA replication-independent nucleosome assembly	4.88e-12
8	tubulin complex assembly (mitotic sister chromatid cohesion sister chromatid segregation)	3.54e-11
9	SCF-dependent proteasomal ubiquitin-dependent protein catabolic process	8.87e-08
10	maintenance of fidelity during DNA-dependent DNA replication; mismatch repair	3.99e-13
11	transcription from RNA polymerase II promote	1.48e-21
12	DNA-dependent DNA replication DNA replication initiation	1.44e-06
13	nucleosome assembly	1.99e-18
14	regulation of transcription during G2/M-phase	1.83e-08
15	DNA repair	2.13e-06
16	M phase of mitotic cell cycle	1.82e-10
17	chromosome segregation	5.87e-10
18	chromatin modification	6.12e-13
19	RNA elongation	1.98e-10
20	transcription from RNA polymerase II promoter	3.61e-21
21	leading strand elongation	9.33e-13

22	nucleosome disassembly chromatin disassembly protein-DNA complex disassembly	4.47e-40
23	maturation of SSU-rRNA	1.56e-18
24	ribosomal small subunit biogenesis	0.005632
25	ubiquitin-dependent protein catabolism	1.49e-16
26	chromatin remodeling	6.46e-13
27	nuclear migration along microtubule	1.62e-06
28	lipid biosynthetic process	6.24e-09
29	mitotic cell cycle spindle assembly checkpoint	0.003157
30	cell morphogenesis	3.53e-09
31	function unknown	N/A
32	regulation of cell division	1.55e-11
33	nuclear mRNA splicing, via spliceosome	1.20e-11
34	Golgi vesicle transport (retrograde vesicle-mediated transport, Golgi to ER)	3.84e-10
35	function unknown	N/A
36	regulation of microtubule polymerization or depolymerization	2.27e-18
37	chromosome segregation	2.45e-10
38	protein amino acid N-linked glycosylation	1.06e-05
39	protein amino acid acetylation	9.87e-12
40	actin cortical patch localization	9.82e-05
41	chromatin modification	3.94e-13
42	mitotic cell cycle (interphase; G1/S transition of mitotic cell cycle)	1.68e-05
43	histone exchange	6.43e-08
44	regulation of cell cycle process	2.60e-12
45	RNA processing	1.08e-05
46	actin cytoskeleton organization	1.71e-05
47	mitochondrion inheritance	1.70e-08
48	exocytosis	2.43e-10
49	function unknown	N/A

50	membrane fusion	1.17e-18
51	retrograde protein transport, ER to cytosol	7.00e-05
52	protein import into nucleus	2.09e-09
53	vacuolar acidification	1.76e-13
54	protein folding	0.00015
55	exocytosis	8.05e-10
56	signal transduction during conjugation with cellular fusion	2.13e-05
57	chromosome segregation	3.68e-07
58	maturation of SSU-rRNA	1.41e-09
59	meiotic cell cycle checkpoint	5.95e-05
60	regulation of mating projection assembly	1.74e-07
61	amino acid metabolic process	0.000343
62	translational initiation	5.81e-08
63	ribosomal protein import into nucleus	3.37e-06
64	nuclear mRNA splicing, via spliceosome	9.55e-05
65	tubulin complex assembly	0.000179
66	amino sugar metabolic process	1.03e-07
67	energy coupled proton transport, down electrochemical gradient	0.002713
68	vesicle-mediated transport	0.000295
69	fatty acid biosynthetic process	0.00162
70	function unknown	N/A
71	vesicle-mediated transport	2.80e-05
72	mitotic sister chromatid segregation	1.74e-08
73	function unknown	N/A
74	nuclear mRNA splicing, via spliceosome	7.89e-05
75	DNA-dependent DNA replication	0.000168
76	nucleosome organization	0.000491
77	regulation of exit from mitosis	1.23e-08
78	DNA repair	2.42e-06
79	regulation of cyclin-dependent protein kinase activity	7.69e-06

80	vesicle-mediated transport	0.002226
81	regulation of mitotic cell cycle	9.57e-05

Table S1 lists functional annotation results of the 82 modules. We annotated functions of the identified modules from biological processes of Gene Ontology and listed the most significant function (p-value<0.01) of each module.
