# Supplementary material for: A novel method to identify cooperative functional modules: study of module coordination in the Saccharomyces cerevisiae cell cycle
Source: BMC Bioinformatics. 2011 Jul 12;12:281. doi: 10.1186/1471-2105-12-281 (PMC3143111; doi:10.1186/1471-2105-12-281)
Supplement: Additional file 8 — Statistical evaluation of the cooperation of the identified module pairs. We evaluated the statistical significance of the cooperation of each module pair identified by our method and listed module pairs that significantly cooperate with genes functional in the cell cycle process or a specific phase. The column Pair_ID lists the unique identifier of each module pair. The column P-value lists the probability that the cooperation of a module pair associates with the cell cycle process or a specific phase. [file 1471-2105-12-281-S8.PDF]

Table S2. Statistical evaluation of the cooperation of the identified module pairs.

Pair_ID[1]	Function (1st)	Function (2nd)	*P*-value(phase)
1 (1,0)	regulation of cell cycle	response to DNA damage stimulus	1.54E-11(cell cycle), 1.74E-05(late G1), 0.00027(G2), 0.0012(M), 0.016(early G1)
2 (3,0)	mitosis	response to DNA damage stimulus	1.03E-14(cell cycle), 7.52E-09(S), 1.16E-0.6(late G1), 0.011(early G1), 0.025(G2)
3 (0,4)	response to DNA damage stimulus	ubiquitin-dependent protein catabolism	0.018(cell cycle)
4 (6,5)	mitochondrial translation	protein insertion into mitochondrial membrane	0.026(cell cycle)
5 (39,0)	protein amino acid acetylation	response to DNA damage stimulus	7.42E-05(cell cycle), 0.00015(G2)
6 (13,0)	nucleosome assembly	response to DNA damage stimulus	1.05E-10(cell cycle), 0.029(G2)
7 (0,7)	response to DNA damage stimulus	DNA replication-indepen dent nucleosome assembly	5.72E-11(cell cycle), 0.0053(late G1)
8 (8,30)	tubulin complex assembly	cell morphogenesis	0.0018(cell cycle)
10 (0,22)	response to DNA damage stimulus	nucleosome disassembly chromatin	2.66E-07(cell cycle), 0.024(late G1), 0.025(S)
11 (54,0)	protein folding	response to DNA damage stimulus	9.32E-08(cell cycle), 0.039(early G1)
12 (13,22)	nucleosome assembly	nucleosome disassembly chromatin	9.82E-08(cell cycle), 0.00068(S), 0.045(M)
13 (0,30)	response to DNA	cell morphogenesis	0(cell cycle),

	damage stimulus		6.68E-11(late G1), 0.020(M)
15 (0,10)	response to DNA damage stimulus	maintenance of fidelity during DNA-dependent DNA replication	0(late G1), 0(cell cycle)
16 (11,0)	transcription from RNA polymerase II promote	response to DNA damage stimulus	0.007(cell cycle)
17 (12,0)	DNA-dependent DNA replication initiation	response to DNA damage stimulus	3.06E-14(cell cycle), 0.00064(late G1), 0.000754(early G1)
18 (13,20)	nucleosome assembly	transcription from RNA polymerase II promoter	0.031(cell cycle)
19 (0,14)	response to DNA damage stimulus	regulation of transcription during G2/M-phase	0(cell cycle), 5.1E-06(M), 0.0027(G2)
20 (0,15)	response to DNA damage stimulus	DNA repair	0(late G1), 0(cell cycle)
21 (16,17)	M phase of mitotic cell cycle	chromosome segregation	0(cell cycle), 1.07E-06(S), 0.014(late G1)
22 (0,18)	response to DNA damage stimulus	chromatin modification	6.93E-05(cell cycle), 0.0010(late G1), 0.0072(S)
25 (0,21)	response to DNA damage stimulus	leading strand elongation	1.1E-11(late G1), 7.23E-11(cell cycle), 0.0032(S)
26 (0,78)	response to DNA damage stimulus	DNA repair	0(cell cycle), 1.75E-11(late G1)
27 (39,22)	protein amino acid acetylation	nucleosome disassembly chromatin	4E-05(cell cycle), 0.00058(S)
28 (28,2)	lipid biosynthetic process	vesicle-mediated transport	0.006(cell cycle), 0.010(late G1)
29 (11,22)	transcription from RNA polymerase II	nucleosome disassembly	0.032(S)

	promote	chromatin	
31 (25,0)	ubiquitin-dependent protein catabolism	response to DNA damage stimulus	0.005(cell cycle)
32 (2,34)	vesicle-mediated transport	Golgi vesicle transport	0.013(cell cycle)
33 (26,22)	chromatin remodeling	nucleosome disassembly chromatin	0.0030(cell cycle), 0.0018(S)
34 (3,27)	mitosis	nuclear migration along microtubule	4.46E-15(cell cycle), 1.71E-06(S), 5.6E-05(M), 0.049(late G1)
35 (29,0)	mitotic cell cycle spindle assembly checkpoint	response to DNA damage stimulus	3.37E-07(cell cycle), 0.000815(early G1), 0.044(G2)
36 (2,31)	vesicle-mediated transport	function unknown	0.0058(late G1), 0.017(cell cycle)
37 (32,0)	regulation of cell division	response to DNA damage stimulus	0(cell cycle), 1.16E-05(late G1), 0.00053(M), 0.0066(early G1)
39 (30,2)	cell morphogenesis	vesicle-mediated transport	0.0020(cell cycle), 0.028(late G1)
40 (44,0)	regulation of cell cycle process	response to DNA damage stimulus	1.15E-14(cell cycle), 5.61E-07(late G1), 0.0038(early G1), 0.0044(M), 0.0075(G2)
42 (36,37)	chromosome segregation	chromosome segregation	5.13E-09(cell cycle), 0.015(late G1), 0.035(S), 0.047(M)
43 (37,17)	chromosome segregation	chromosome segregation	0(cell cycle), 4.07E-08(S), 0.0017(late G1), 0.014(G2)
44 (38,2)	protein amino acid N-linked glycosylation	vesicle-mediated transport	0.033(cell cycle)

45 (13,39)	nucleosome assembly	protein amino acid acetylation	3.09E-05(cell cycle), 0.014(S)
47 (30,14)	cell morphogenesis	regulation of transcription during G2/M-phase	0(cell cycle), 2.3E-08(M), 0.010(G2)
49 (13,41)	nucleosome assembly	chromatin modification	1.52E-11(cell cycle), 0.00022(S), 0.018(M)
50 (42,30)	mitotic cell cycle (interphase; G1/S transition of mitotic cell cycle)	cell morphogenesis	0(cell cycle), 6.4E-07(late G1), 0.00014(M), 0.00174(early G1), 0.0029(G2), 0.040(S)
51 (36,17)	regulation of microtubule polymerization or depolymerization	chromosome segregation	2.81E-11(cell cycle), 6.15E-05(S), 0.0065(late G1), 0.027(M)
52 (39,43)	protein amino acid acetylation	histone exchange	0.0018(cell cycle)
54 (46,47)	actin cytoskeleton organization	mitochondrion inheritance	0.043(cell cycle)
55 (48,30)	exocytosis	cell morphogenesis	3.92E-07(cell cycle), 0.00033(late G1), 0.046(early G1)
56 (49,2)	function unknown	vesicle-mediated transport	0.03(cell cycle)
57 (50,2)	membrane fusion	vesicle-mediated transport	0.0022(cell cycle)
58 (51,28)	retrograde protein transport, ER to cytosol	lipid biosynthetic process	0.027(cell cycle)
59 (0,52)	response to DNA damage stimulus	protein import into nucleus	0.0015(late G1), 0.0054(M), 0.0092(cell cycle)
60 (28,49)	lipid biosynthetic process	function unknown	0.03(cell cycle)
62 (3,30)	mitosis	cell morphogenesis	5.51E-07(cell cycle), 0.0038(late G1), 0.042(G2)

63 (30,54)	cell morphogenesis	protein folding	8.32E-07(cell cycle), 0.018(early G1)
64 (32,55)	regulation of cell division	exocytosis	6.92E-07(cell cycle), 0.0012(late G1)
65 (56,0)	signal transduction during conjugation with cellular fusion	response to DNA damage stimulus	0.027(cell cycle)
66 (30,57)	cell morphogenesis	chromosome segregation	1.21E-08(cell cycle)
67 (45,58)	RNA processing	maturation of SSU-rRNA	0.025(cell cycle)
68 (0,59)	response to DNA damage stimulus	meiotic cell cycle checkpoint	0.0035(cell cycle), 0.037(M)
69 (48,60)	exocytosis	regulation of mating projection assembly	2.66E-07(cell cycle), 0.00095(late G1), 0.0023(early G1)
74 (54,3)	protein folding	mitosis	0.012(cell cycle)
76 (13,32)	nucleosome assembly	regulation of cell division	4.2E-08(cell cycle), 0.019(late G1)
77 (66,46)	amino sugar metabolic process	actin cytoskeleton organization	9.75E-05(cell cycle)
80 (69,2)	fatty acid biosynthetic process	vesicle-mediated transport	0.015(cell cycle), 0.034(late G1)
83 (3,72)	mitosis	mitotic sister chromatid segregation	4.28E-12(cell cycle), 0.00089(late G1), 0.0086(S), 0.013(M)
85 (28,0)	lipid biosynthetic process	response to DNA damage stimulus	0.017(cell cycle)
86 (30,73)	cell morphogenesis	function unknown	0.00063(cell cycle), 0.018(M), 0.025(late G1)
88 (75,76)	DNA-dependent DNA replication	nucleosome organization	2.81E-11(cell cycle), 1.7E-06(late G1)
89 (77,0)	regulation of exit from mitosis	response to DNA damage stimulus	1.15E-06(cell cycle), 0.0028(G2), 0.0029(M)
90 (78,10)	DNA repair	maintenance of fidelity during	0(cell cycle), 4.27E-11(late G1)

		DNA-dependent DNA replication; mismatch repair	
91 (45,14)	RNA processing	regulation of transcription during G2/M-phase	1.01E-07(cell cycle), 0.016(G2), 0.04(M)
92 (3,79)	mitosis	regulation of cyclin-dependent protein kinase activity	3.72E-15(cell cycle), 2.95E-08(M), 0.0033(S), 0.0056(G2), 0.010(early G1), 0.028(late G1)
93 (30,32)	cell morphogenesis	regulation of cell division	0(cell cycle), 6.25E-0.5(late G1), 8.91E-05(M), 0.000168(early G1), 0.023(S)
94 (0,80)	response to DNA damage stimulus	vesicle-mediated transport	0.044(cell cycle)
95 (2,14)	vesicle-mediated transport	regulation of transcription during G2/M-phase	1.19E-11(cell cycle), 0.0066(G2)
96 (30,52)	cell morphogenesis	protein import into nucleus	0.0054(cell cycle), 0.013(M), 0.028(early G1), 0.044(late G1)
97 (48,44)	exocytosis	regulation of cell cycle process	0.0022(cell cycle), 0.003(M), 0.011(late G1), 0.047(G2)
98 (0,73)	response to DNA damage stimulus	function unknown	0.00079(cell cycle), 0.037(M)
99 (56,73)	signal transduction during conjugation with cellular fusion	function unknown	0.0015(cell cycle), 0.014(late G1)
100 (1,81)	regulation of cell cycle	regulation of mitotic cell cycle	9.94E-12(cell cycle), 1.22E-05(M), 1.42E-05(G2)

[1]Pair_ID indicates the unique identifier of each identified module pair; (ID_1, ID_2): indicates the unique identifiers (IDs) of the first and second modules of a module pair, respectively.

We evaluated the statistical significance of the cooperation of each module pair identified by our method and listed module pairs that significantly cooperate with genes functional in the cell cycle process or a specific phase (p-value < 0.05). The column ***P*-value** lists the probability that the cooperation of a module pair associates with the cell cycle process or a specific phase.
