# Supplementary material for: A novel method to identify cooperative functional modules: study of module coordination in the Saccharomyces cerevisiae cell cycle
Source: BMC Bioinformatics. 2011 Jul 12;12:281. doi: 10.1186/1471-2105-12-281 (PMC3143111; doi:10.1186/1471-2105-12-281)
Supplement: Additional file 10 — Cooperative relationship media ted by Cdc28 and phase-related regulators. Additional file 10 lists cooperative module pairs that cooperate with essential regulators of the yeast cell cycle. The column Regulator lists regulators cooperating with a module pair. [file 1471-2105-12-281-S10.PDF]

Table S3. Cooperative relationships mediated by Cdc28 and phase-related regulators.

Pair_ID[1]	Function (1st)	Function (2nd)	Regulator (ID)
1 (1,0)	regulation of cell cycle	response to DNA damage stimulus	*CLN3, CLB3, MBP1, CLN1, STB1, NDD1*
2 (3,0)	mitosis	response to DNA damage stimulus	*MBP1, SWI6, FKH2, CDC21*
3 (0,4)	response to DNA damage stimulus	ubiquitin-dependent protein catabolism	*FKH1*
5 (39,0)	protein amino acid acetylation	response to DNA damage stimulus	*CDC36, CDC7*
6 (13,0)	nucleosome assembly	response to DNA damage stimulus	*CDC24, MBP1, SWI4, FKH1, SWI6*
7 (0,7)	response to DNA damage stimulus	DNA replication-independent nucleosome assembly	*CDC9*
8 (8,30)	tubulin complex assembly	cell morphogenesis	*SWI6, MCM1*
9 (9,4)	SCF-dependent proteasomal ubiquitin-dependent protein catabolic process	ubiquitin-dependent protein catabolism	*CDC39*
12 (13,22)	nucleosome assembly	nucleosome disassembly chromatin	*CDC24*
13 (0,30)	response to DNA damage stimulus	cell morphogenesis	*MBP1, SWI4, SWI6, STB1, NDD1*
15 (0,10)	response to DNA damage stimulus	maintenance of fidelity during DNA-dependent DNA replication	*MBP1, CDC7, FKH1*
16 (11,0)	transcription from RNA polymerase II promote	response to DNA damage stimulus	*SWI6*

17 (12,0)	DNA-dependent DNA replication initiation	response to DNA damage stimulus	*CDC7*
19 (0,14)	response to DNA damage stimulus	regulation of transcription during G2/M-phase	*CLB3, MBP1, CDC7, CDC14, FKH1, ACE2, CDC5, MCM1, STB1, FKH2, NDD1*
20 (0,15)	response to DNA damage stimulus	DNA repair	*FKH1*
22 (0,18)	response to DNA damage stimulus	chromatin modification	*SWI4, SWI6, CDC3, STB1, FKH2, CDC21*
33 (26,22)	chromatin remodeling	nucleosome disassembly chromatin	*SWI5*
34 (3,27)	mitosis	nuclear migration along microtubule	*CLB3, CLB4*
35 (29,0)	mitotic cell cycle spindle assembly checkpoint	response to DNA damage stimulus	*CDC5*
37 (32,0)	regulation of cell division	response to DNA damage stimulus	*CDC3, SWI4, SWI6, CLN1, STB1, NDD1, CLN2*
38 (0,33)	response to DNA damage stimulus	nuclear mRNA splicing, via spliceosome	*MBP1*
39 (30,2)	cell morphogenesis	vesicle-mediated transport	*STB1*
40 (44,0)	regulation of cell cycle process	response to DNA damage stimulus	*CLB3, STB1, FKH2, NDD1*
41 (35,28)	function unknown	lipid biosynthetic process	*SWI5*
44 (38,2)	protein amino acid N-linked glycosylation	vesicle-mediated transport	*SWI5*
45 (13,39)	nucleosome assembly	protein amino acid acetylation	*SWI6, MCM1*

46 (39,11)	protein amino acid acetylation	transcription from RNA polymerase II promote	*CDC39, SWI6, MCM1*
47 (30,14)	cell morphogenesis	regulation of transcription during G2/M-phase	*CDC28, CLB3, MBP1, SWI5, FKH1, ACE2,, CLB4, CDC5, MCM1, STB1, FKH2, NDD1*
49 (13,41)	nucleosome assembly	chromatin modification	*MCM1, FKH2*
50 (42,30)	mitotic cell cycle (interphase; G1/S transition of mitotic cell cycle)	cell morphogenesis	*MBP1, SWI5, SWI4, FAR1, ACE2, SWI6, CLN1, STB1, NDD1, CLN2*
52 (39,43)	protein amino acid acetylation	histone exchange	*CDC36, SWI4*
55 (48,30)	exocytosis	cell morphogenesis	*CDC24, MBP1, SWI4, FKH1, FAR1, CLN2*
58 (51,28)	retrograde protein transport, ER to cytosol	lipid biosynthetic process	*NDD1*
62 (3,30)	mitosis	cell morphogenesis	*MBP1, SWI6, STB1*
63 (30,54)	cell morphogenesis	protein folding	*CDC37, SWI4*
64 (32,55)	regulation of cell division	exocytosis	*CDC24, MBP1, FAR1, CLN2*
65 (56,0)	signal transduction during conjugation with cellular fusion	response to DNA damage stimulus	*CLB1, CLN1, CLN3*
66 (30,57)	cell morphogenesis	chromosome segregation	*MBP1, SWI6, MCM1, CLN1, FKH2*
68 (0,59)	response to DNA damage stimulus	meiotic cell cycle checkpoint	*SWI5*
69 (48,60)	exocytosis	regulation of mating projection assembly	*CDC24, FUS3, SWI4, FAR1*

74 (54,3)	protein folding	mitosis	*SWI4*
76 (13,32)	nucleosome assembly	regulation of cell division	*CDC24, SWI4, SWI6, MCM1, FKH2, CLN2*
79 (71,68)	vesicle-mediated transport	vesicle-mediated transport	*FKH1*
81 (70,4)	function unknown	ubiquitin-dependent protein catabolism	*CDC11*
82 (2,71)	vesicle-mediated transport	vesicle-mediated transport	*FKH1*
83 (3,72)	mitosis	mitotic sister chromatid segregation	*MBP1, SWI6, CDC5*
85 (28,0)	lipid biosynthetic process	response to DNA damage stimulus	*CDC7, SWI4*
86 (30,73)	cell morphogenesis	function unknown	*STB1*
88 (75,76)	DNA-dependent DNA replication	nucleosome organization	*CDC7*
89 (77,0)	regulation of exit from mitosis	response to DNA damage stimulus	*CDC24, SWI4, CLB1, SWI6, CDC3, CDC5, STB1*
91 (45,14)	RNA processing	regulation of transcription during G2/M-phase	*CDC14, FKH1, ACE2*
92 (3,79)	mitosis	regulation of cyclin-dependent protein kinase activity	*CLB3, MBP1, CLB1, ACE2, SWI6, CLB4, STB1, FKH2*
93 (30,32)	cell morphogenesis	regulation of cell division	*MBP1, SWI4, FAR1, SWI6, MCM1, CLN1, STB1, NDD1, CLN2*
94 (0,80)	response to DNA damage stimulus	vesicle-mediated transport	*CDC7*
95 (2,14)	vesicle-mediated transport	regulation of transcription during G2/M-phase	*SWI5, FKH1, MCM1, STB1, FKH2*

96 (30,52)	cell morphogenesis	protein import into nucleus	*SWI4*
97 (48,44)	exocytosis	regulation of cell cycle process	*MBP1, FKH1, FAR1, CLN2*
98 (0,73)	response to DNA damage stimulus	function unknown	*STB1*
100 (1,81)	regulation of cell cycle	regulation of mitotic cell cycle	*CDC15, CDC28, CLB3, CDC5, CLB2*

[1]Pair_ID indicates the unique identifier of each identified module pair; (ID_1, ID_2): indicates the unique identifiers (IDs) of the first and second modules of a module pair, respectively.

Table S3 lists cooperative module pairs that cooperate with essential regulators of the yeast cell cycle (Cdc28, cyclins, CDC genes, and phase-specific transcription factors). The column **Regulator** lists regulators cooperating with a module pair.
