# Supplementary material for: A novel method to identify cooperative functional modules: study of module coordination in the Saccharomyces cerevisiae cell cycle
Source: BMC Bioinformatics. 2011 Jul 12;12:281. doi: 10.1186/1471-2105-12-281 (PMC3143111; doi:10.1186/1471-2105-12-281)
Supplement: Additional file 11 — Modules containing Cdc28 and phase-related regulators. We listed modules identified by our method that contain Cdc28 and phase-related regulators. [file 1471-2105-12-281-S11.PDF]

Table S4. Modules containing Cdc28 and phase-related regulators.

Phase	Regulator	Module ID
G1, S	*SWI4*	30, 66
G1	*STB1*	32, 42
G1, S	*SWI6*	0
G1, M	*ACE2*	14
G1, S	*SKN7*	0
G1	*CLN3*	32
G1	*FUS3*	32
G1	*CLN2*	32, 42, 79
G1, G2, M	*CDC28*	1, 30, 32, 42, 44, 79
S	*CDC24*	77
S	*CDC7*	12
S, G2	*NDD1*	14
G2	*FKH1*	14
G2	*FKH2*	14
G2, M	*CLB2*	0
G2	*CDC15*	44
G2	*CDC5*	44
G2	*CDC14*	44
G2, M	*CLB3*	3
M	*SWI5*	14
